# Stability of cytokine, cellular and clinical response to the intravenous LPS challenge repeated after one year: a healthy volunteer trial

**DOI:** 10.1007/s00430-025-00823-5

**Published:** 2025-03-06

**Authors:** Anselm Jorda, Lena Pracher, Sabine Eberl, Alina Nussbaumer-Pröll, Maysa Sarhan, Maria Weber, Markus Wahrmann, Valentin al Jalali, Felix Bergmann, Marlene Prager, Amelie Leutzendorff, Maria Sanz-Codina, Lara Tegrovsky, Theresa Pecho, Bernd Jilma, Lena Müller, Andreas Spittler, Marianne Rocha-Hasler, Julia Eckl-Dorna, Anna Kusienicka, Matthias Farlik, Markus Zeitlinger

**Affiliations:** 1 Department of Clinical Pharmacology, Medical University of Vienna, Waehringer Guertel 18-20, Vienna, 1090 Austria; 2 Division of Nephrology and Dialysis, Department of Internal Medicine III, Vienna, Austria; 3 Division of Infectious Diseases and Tropical Medicine, Department of Medicine III, Vienna, Austria; 4 Core Facilities, Medical University of Vienna, Vienna, Austria; 5 Research Laboratories, Core Facility Flow Cytometry & Department of Surgery, Medical University of Vienna, Vienna, Austria; 6 Department of Otorhinolaryngology, Medical University of Vienna, Vienna, Austria; 7 Department of Dermatology, Medical University of Vienna, Vienna, Austria

**Keywords:** Endotoxin, Endotoxemia, Sepsis, Inflammation, Innate immune system

## Abstract

Whether the magnitude of individual cytokine, cellular, and clinical responses to the intravenous lipopolysaccharide (LPS) challenge is constant in individuals over extended time periods is unknown. Nine healthy volunteers received an intravenous LPS injection of 2 ng/kg bodyweight twice at intervals of at least one year. Circulating cytokines and leukocyte subsets were quantified using a multiplex immunoassay and cytometry by time-of-flight, respectively. Self-reported symptoms and vital signs were also assessed. We observed moderate to strong intra-individual correlations in the responsiveness of most cytokines (IL-6 [AUC_0 − 10_]: *R* = 0.93, *p* < 0.001; CRP [mg/dL]: *R* = 0.88, *p* = 0.004; IL-8 [AUC_0 − 10_]: *R* = 0.71, *p* = 0.031; TNF-alpha [AUC_0 − 10_]: *R* = 0.67, *p* = 0.047; IL-10 [AUC_0 − 10_]: *R* = 0.42, *p* = 0.26) and cellular subsets (CD8 T lymphocytes: *R* = 0.9, *p* = 0.002; B lymphocytes [G/L]: *R* = 0.89, *p* = 0.003; CD4 T lymphocytes: *R* = 0.84, *p* = 0.001; neutrophils: *R* = 0.80, *p* = 0.017; monocytes: *R* = 0.16, *p* = 0.710) between the 1st and 2nd LPS challenges. Vital signs and symptoms were not reproducible. While the average cellular and clinical response was similar between the two LPS challenges, we found a significantly attenuated AUC_0 − 10_ of IL-6 (percent difference, -41.9% [95% CI -73.0 – -10.7]) and TNF-alpha (percent difference, -35.7% [95% CI -70.0 – -1.6]) at the 2nd LPS challenge. Individual cytokine and cellular responses to intravenous LPS showed a significant degree of correlation when measured more than one year apart. These correlations did not translate to the reproducibility of clinical symptoms and vital signs, which showed greater variability and were not constant over time. The partly reduced cytokine release in the 2nd LPS challenge might be interpreted as an indicator of a long-lasting tolerance to endotoxin.

**Supplementary Information:**

The online version contains supplementary material available at 10.1007/s00430-025-00823-5.

## Introduction

The intravenous endotoxin or lipopolysaccharide (LPS) challenge is an established model for investigating various aspects of the human innate immune system in healthy volunteers [1]. In a previous study, we demonstrated the stability of the cytokine response to repeated ex vivo whole blood LPS stimulation [2]. However, the long-term stability of cytokine, cellular, and clinical responses to repeated intravenous LPS challenges remains unknown. Specifically, it is unclear to what extent LPS responsiveness reflects stable, intrinsic characteristics of an individual – such as genetic traits or sex – or if it is predominately modulated by time-varying environmental factors, such as stress, seasonal variation, infections, vaccinations, or medication. In recent decades, it has been increasingly recognized that the ‘innate’ immune system, which has historically been described as incapable of learning, also has a certain memory capacity [3], a process that Netea and his colleagues have termed ‘trained immunity’ [4]. 

The present study had two key objectives. First, we aimed to determine whether there is intra-individual reproducibility in the molecular, cellular, and clinical responses to intravenous LPS over an extended period. Second, we sought to explore whether the immune response decreases with repeated LPS exposure, a phenomenon commonly referred to as endotoxin tolerance [5]. 

In this study, healthy volunteers received intravenous LPS on two occasions, separated by over a year – considerably longer than previous studies, which typically examined reproducibility over a few weeks [6, 7]. By investigating whether the immune response remains constant over this extended period of time, we aim to provide new insights into the long-term stability and memory of the innate immune system.

## Materials and methods

### Study design and participants

In this clinical study, 12 healthy volunteers (6 females and 6 males) participated in the intravenous endotoxin challenge. These subjects were subsequently invited to undergo a 2nd challenge 1 to 1.5 years later. The key inclusion criteria were physical and mental health and an age between 20 and 30 years. The key exclusion criteria were nicotine use, history of substance abuse, concomitant medication other than birth control, weight > 95 kg, weight < 60 kg for men or < 50 kg for women, any vaccination within 4 weeks before the first study day, and previous participation in an intravenous LPS study. The exact inclusion and exclusion criteria are presented in Supplementary Table 1.

The conduct of this study followed the principles of Good Clinical Practice and the Declaration of Helsinki. The Ethics Committee of the Medical University of Vienna approved the study (ID 1676/2020). Oral and written consent to participate in the study was collected from all subjects before inclusion.

### Intravenous endotoxin challenge

Details of the intravenous endotoxin challenge have been described previously [8]. All subjects underwent the 2nd human endotoxin challenge at least one year after the 1st challenge. LPS was administered as a single intravenous dose of 2 ng/kg body weight over 1 min. Participants were clinically monitored by continuous or frequent assessment of body temperature, blood pressure, heart rate, oxygen saturation, and ECG for up to 6 h after LPS infusion. All participants received an 8-hour infusion of 0.9% saline solution at an infusion rate of 100 mL/h to prevent hypotension and dehydration. Antipyretics were not routinely administered. On request, subjects could receive 1 g of paracetamol intravenously to relieve symptoms, which has been shown to not influence the cytokine response [9]. Supplementary Table 2 lists the lifestyle restrictions before the study days, which were implemented to reduce heterogeneity and sources of variability.

### Sampling and data collection

Plasma sampling for cytokine determination was performed at baseline (0 h) and 0.5, 1, 1.5, 2, 4, and 10 h after LPS infusion. Blood sampling for the cellular quantification using cytometry by time-of-flight (CyTOF) was performed at baseline (0 h) and 4 h after LPS infusion. To quantify the clinical response, participants were asked to grade the severity of solicited adverse events (nausea, headache, chills, and myalgia [10]) from 0 to 10 every 30 min for up to 6 h after LPS infusion. Likewise, vital signs (blood pressure, heart rate, and body temperature) were assessed every 30 min for up to 6 h after LPS infusion.

### Cytokine quantification with a multiplex immunoassay

A multiplex immunoassay was used to quantify circulating interleukin-6 (IL-6), interleukin-8 (IL-8), interleukin-10 (IL-10), and tumor necrosis factor-alpha (TNF-alpha) serum concentrations. The Human Luminex^®^ Discovery immunoassay from R&D Systems is a magnetic microparticle-based immunoassay using an immobilized capture antibody and labeled detection antibody similar to traditional sandwich ELISA. Details on this method have been described before [11]. Measurements were performed in duplicates. The lower limits of quantification were as follows: IL-10, 36.2 pg/mL; IL-8, 1.5 pg/mL; IL-6, 9.5 pg/mL; and TNF-alpha, 7.3 pg/mL.

Supplementary Table 3 shows the performance of the repeated measurement of the low- and high-concentration quality control (QC) samples. The low-concentration QC samples had coefficients of variation below 15%. The high-concentration QC samples showed a coefficient of variation below 20%, except for IL-8 (35%). Supplementary Fig. 1 shows the linearity of the standard curves for the individual cytokines. Because C-reactive protein (CRP) shows a delayed increase in plasma, compared to the fast release of cytokines, it was assessed once at 24 h after LPS administration by the routine laboratory tests of the hospital.

### Cellular quantification with mass cytometry

Mass cytometry by time-of-flight (CyTOF^®^) was used to quantify the relative frequency of leukocyte populations and to characterize subsets from whole blood. For staining, whole blood samples were processed and incubated with the Maxpar^®^ DirectTM Immune Profiling Assay (Standard BioTools). This system uses a 30-marker metal-tagged antibody panel in a dry-single tube format to enable the identification of 36 immune cell subsets. Data acquisition (target: 200,000 events per sample) was performed on the HeliosTM mass cytometer (StandardBioTools). Sample processing and data acquisition strictly followed the manufacturer’s protocols using the recommended reagents.

After acquisition with the Helios instrument, samples were normalized with the CyTOF software version 7.0 (Standard BioTools). Normalized FCS files were uploaded to the Cytobank platform (version 10.5) (Beckman Coulter Inc., CA) [12]. After clean-up, live cells were uniformly gated further to obtain the relative abundance of 36 distinct subsets as described in Supplementary Table 4. The absolute leukocyte counts at baseline and at 4 h were measured by the routine laboratory tests of the hospital.

### Data analysis

Baseline characteristics were reported descriptively using mean and standard deviation (SD) and numbers with percentages (n [%]). Serum cytokine concentrations were analyzed based on the area under the curve from baseline to 10 h after LPS infusion (AUC_0 − 10_, pg*h/mL) for each cytokine using the trapezoidal formula. The abundance of circulating leukocyte subsets is presented as absolute concentration (G/L), relative to total leukocyte count (%), and relative to total subpopulation (e.g., % of total B cells). Differences in cytokine AUCs_0 − 10_, C-reactive protein concentration, and absolute and relative frequencies of leukocyte subsets (Δ baseline to 4 h) were tested using the paired sample T-test. The correlation of cytokine AUCs_0 − 10_, changes in relative and absolute leukocyte subsets, and highest levels of vital signs and symptom severity between the 1st and 2nd LPS challenge was calculated using the Pearson correlation coefficient. We also assessed the correlation between the individual cytokine AUCs_0 − 10_ and the mean cytokine AUC ranks calculated by averaging the ranks for IL-8, IL-10, IL-6 and TNF-alpha for each subject. As previously suggested, Pearson correlation coefficients *R* < 0.4 indicated low, 0.4 to 0.7 moderate, 0.7 to 0.9 high, and > 0.9 very high correlation [13]. An analysis of variance (ANOVA) was performed to explore the interaction between the LPS challenge (1st or 2nd ) and the concentration-time profile of the individual cytokines. Peak concentrations (C_max_) and time to reach the peak concentrations (T_max_) were also compared between the 1st and 2nd LPS challenge. No traditional sample size calculation was performed. P values below 0.05 were considered statistically significant. Because of the exploratory nature of this study no adjustments for multiple comparisons were performed. Statistical analysis and visualization were performed using R (version 4.1.2, 2021, Vienna, Austria) and RStudio (RStudio Team, 2020).

## Results

### Participants

Of the 12 subjects who underwent the 1st LPS challenge, 3 declined to participate in the 2nd LPS challenge, leaving 9 subjects (4 females, 5 males) for the final analysis. The baseline characteristics are shown in Table 1. The mean (SD) age at the 1st endotoxin challenge was 24 (3) years. As expected, males had a greater body height (mean [SD] cm, 186 [7] vs. 170 [7]) and weight (mean [SD] kg, 80 [11] vs. 67 [13]) than females, but a similar body mass index (mean [SD] kg/m^2^, 23 [3] vs. 23 [5]). The mean (SD) time from the 1st to the 2nd endotoxin challenge was 63 (1) weeks. The mean (SD) total dose of intravenous LPS was 148 (27) ng/kg bodyweight at the 1st and 149 (28) ng/kg bodyweight at the 2nd endotoxin challenge. A previous episode of symptomatic COVID-19 was reported by 7 (78%) of the 9 participants. All subjects received at least one SARS-CoV-2 vaccine before the study.

**Table 1:** Baseline characteristics of included subjects

	Overall	Females	Males
N=	9	4	5
Age at 1st LPS challenge, years (mean [SD])	23.7 (3.0)	21.8 (2.2)	25.2 (2.8)
Height, cm (mean [SD])	179.0 (10.3)	170.3 (6.6)	186.0 (6.5)
Weight at 1st LPS challenge, kg (mean [SD])	74.1 (12.8)	67.4 (12.7)	79.6 (11.0)
BMI at 1st LPS challenge, kg/m2 (mean [SD])	23.1 (3.6)	23.28 (4.7)	23.02 (3.1)
LPS dose at 1st LPS challenge, ng (mean [SD])	147.6 (26.9)	132.5 (26.3)	159.6 (22.8)
Weight at 2nd LPS challenge, kg (mean [SD])	75.1 (14.0)	70.1 (17.5)	79.2 (10.7)
BMI at 2nd LPS challenge, kg/m2 (mean [SD])	23.5 (4.4)	24.2 (6.3)	22.9 (2.9)
LPS dose at 2nd LPS challenge, ng (mean [SD])	148.9 (27.7)	138.8 (34.0)	157.0 (22.)
Weeks between 1st and 2nd LPS challenge (mean [SD])	62.6 (10.5)	62.1 (8.4)	63.0 (12.9)
Any Allergies (n [%])	2 (22.2)	1 (25.0)	1 (20.0)
Previous Covid-19 (n [%])	7 (77.8)	3 (75.0)	4 (80.0)
Oral contraception (n [%])	NA	3 (75.0)	NA

Data on cytokines and clinical symptoms were available from all 9 subjects. Cellular data obtained using CyTOF and the CRP value after 24 h were available for 8 of the 9 subjects included (one subject could not be analyzed because of an pre-analytical error of the sample).

### Cytokine and C-reactive protein response to LPS

**Fig. 1:**
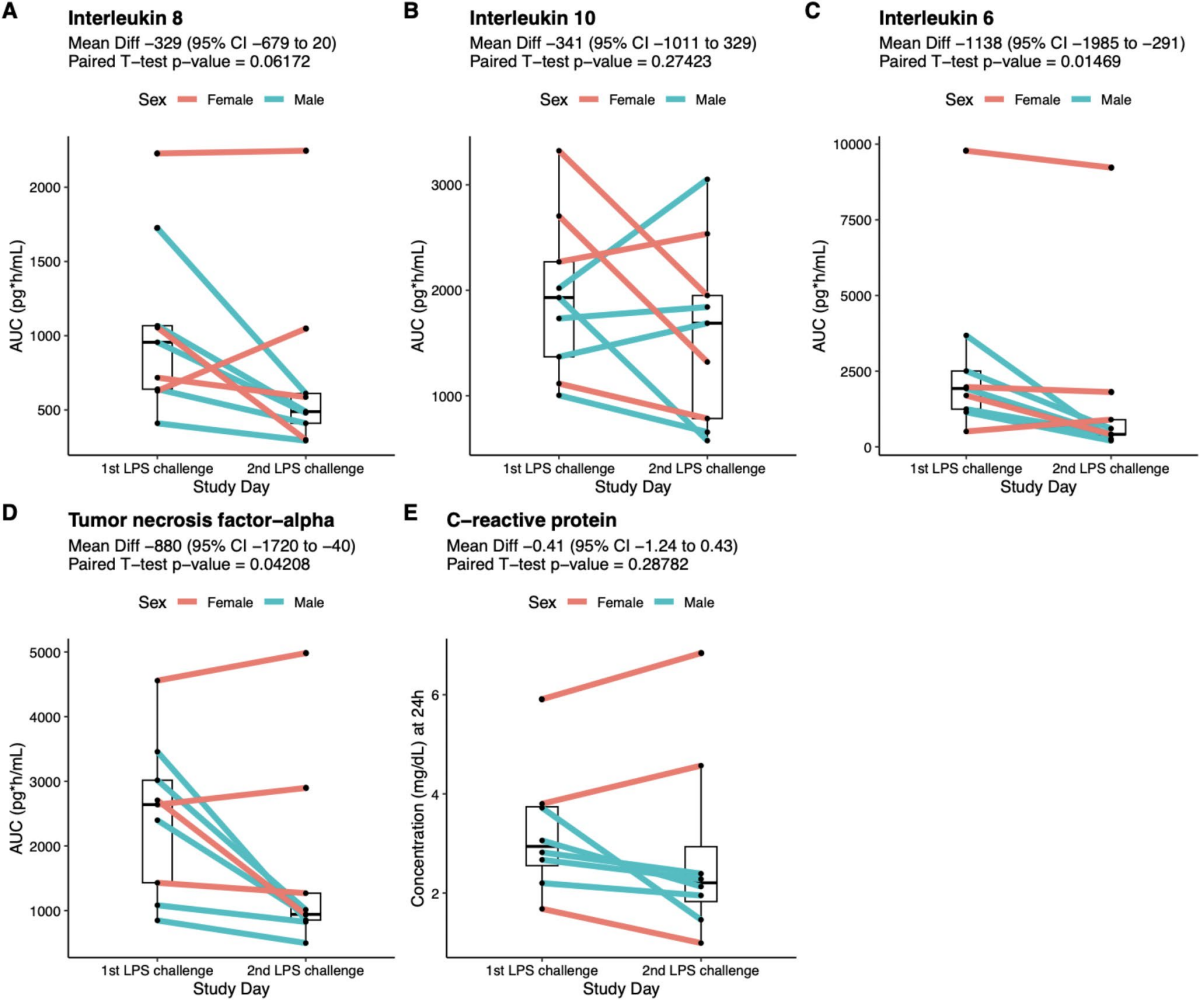
Comparison of between the individual cytokine areas under the curve (AUCs_0 − 10_) and 24 h C-reactive protein levels of the 1st and 2nd endotoxin challenge. (**A**) Interleukin 8, (**B**) Interleukin 10), (**C**) Interleukin 6, (**D**) Tumor necrosis factor-alpha, and (**E**) C-reactive protein. AUCs_0 − 10_ are based on the concentration-time profiles from baseline up to 10 h after LPS infusion. Each figures includes the results of the paired-sample t test between he 1st and 2nd LPS challenge. Colors indicate the sex of the individuals

Individual concentration-time profiles of IL-6, IL-10, IL-6, and TNF-alpha during the 1st and 2nd LPS challenge are depicted in Supplementary Fig. 2. The AUC_0 − 10_ of the individual cytokine responses and the 24-hour concentration of CRP after the 1st and 2nd LPS challenges are shown in Fig. 1 and Supplementary Table 5. Mean AUC_0 − 10_ of cytokines and the 24-hour concentration of CRP was higher after the 1st LPS challenge than after the 2nd LPS challenge, but a statistically significant difference was only observed for IL-6 (mean [SD] AUC_0 − 10_, 2719 [2798] vs. 1581 [2909] pg*h/mL; mean difference − 1138 [95% CI -1985 to -291], percent difference − 41.9% [95% CI -73.0 to -10.7], *p* = 0.015) and for TNF-alpha (2459 [1191] vs. 1579 [1450] pg*h/mL; mean difference − 880 [95% CI -1720 to -40], percent difference − 35.7% [95% CI -70.0 to -1.6], *p* = 0.042) (Fig. 1). Using ANOVAs, a statistically significant interaction between the sequence of LPS challenge (1st or 2nd ) and the concentration per timepoint was found for IL-8 (F = 2.37, *p* = 0.037) and TNF-alpha (F = 4.09, *p* = 0.001) but not for IL-10 and IL-6 (Supplementary Table 6). Supplementary Table 7 provides the comparisons of C_max_ and T_max_ between the 1st and 2nd LPS challenge for each cytokine. A comparison of AUC, C_max_, and T_max_ values between male and female subjects is shown in Supplementary Table 8.

**Fig. 2:**
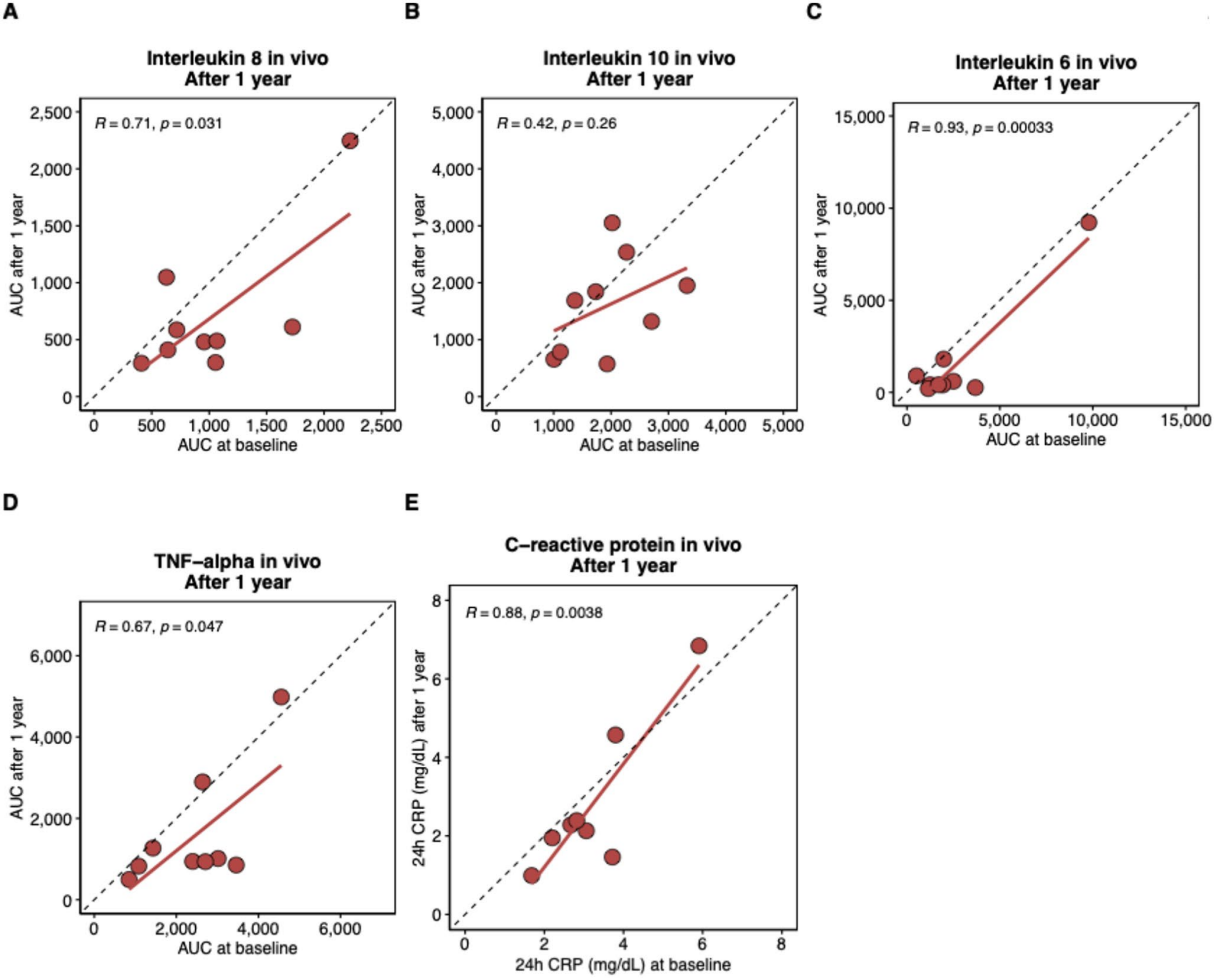
Dot plots assessing the correlation between the individual cytokine AUCs_0 − 10_ and 24 h C-reactive protein levels of the 1st and 2nd endotoxin challenge. (**A**) Interleukin 8, (**B**) Interleukin 10), (**C**) Interleukin 6, (**D**) Tumor necrosis factor-alpha, and (**E**) C-reactive protein. The red line is the regression line based on the observed AUCs_0 − 10_. Each dot represents one participant. The dashed line indicates a perfect correlation. R is the Pearson correlation coefficient, accompanied by unadjusted p values

Figure 2 shows the intra-individual correlation of cytokine and CRP responses between the 1st and 2nd LPS challenge. Moderate to very strong correlations between the 1st and 2nd LPS challenge were observed for the individual AUCs_0 − 10_ of IL-8 (*R* = 0.71, *p* = 0.031), IL-6 (*R* = 0.93, *p* < 0.001), TNF-alpha (*R* = 0.67, *p* = 0.047) and the 24 h concentration of CRP (*R* = 0.88, *p* = 0.004). IL-10 showed no significant correlation (*R* = 0.42, *p* = 0.260) (Fig. 2). As shown in Supplementary Fig. 3, TNF-alpha showed the highest level of correlation with the mean AUC rank during the 1st (*R* = 0.91, *p* = 0.00054) and the 2nd LPS challenge (*R* = 0.77, *p* = 0.014). IL-10 did not correlate with the mean AUC rank of all four cytokines (Supplementary Fig. 3). Supplementary Fig. 4 shows the correlation coefficients between the individual cytokine AUCs observed during the 1st and 2nd LPS challenge.

### Cellular response to LPS

**Fig. 3:**
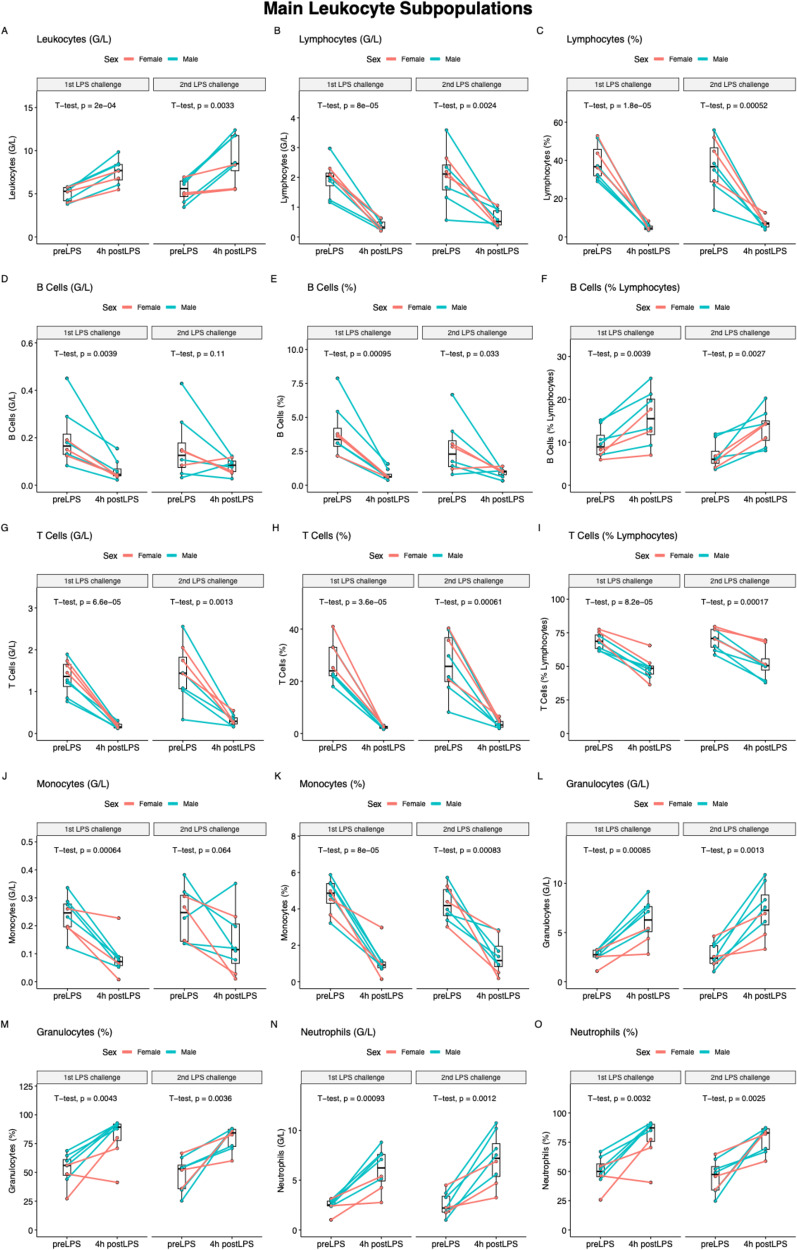
Main leukocyte subpopulations before and 4 h after LPS administration at the 1st and 2nd LPS challenge. Depicted are absolute (G/L) and relative (% of all leukocytes or % of the population) cell frequencies quantified by CyTOF. Because CyTOF quantification was unsuccessful in one female subject, only cell data of 8 of the 9 subjects are available

Figure 3 depicts the dynamics of the main leukocyte populations after LPS infusion. At 4 h after LPS infusion, a significant increase in leukocytes was observed, which was mainly due to absolute and relative neutrophilia. Meanwhile, a significant absolute and relative decrease in mononuclear cells (lymphocytes and monocytes) was observed (Fig. 3). Overall, the mean changes (Δ baseline to 4 h) in absolute and relative frequencies of subsets were largely similar and showed no statistical difference between the 1st and 2nd LPS challenges. The few exceptions showing a statistically significant difference are depicted in Supplementary Fig. 5 to 7.

More details on the complex dynamics of subsets of B lymphocytes, CD4 T lymphocytes, CD8 T lymphocytes, helper T cells, regulatory T cells, natural killer T cells, gamma-delta T cells, monocytes, dendritic cells, natural killer cells, and granulocytes are depicted in Supplementary Fig. 8 to 16. LPS administration had a significant impact on the relative and absolute abundance of almost all leukocyte subpopulations.

The decrease in B cells was mainly due to a relative decrease in memory B cells, with the proportion of naïve and plasma B cells being constant (Supplementary Fig. 8).

The decrease in CD4 and CD8 T cells was due to a decrease in all subpopulations (i.e., naïve, central memory, effector memory, and terminal effector cells) (Supplementary Figs. 9 and 10). A significant decrease was also observed for regulatory T cells, helper T cells, natural killer T cells, and gamma delta T cells (Supplementary Figs. 11 and 12). Within the overall decrease in monocytes, there was a relative increase in the proportion of classical monocytes (within all monocytes) and a decrease in the proportion of transitional and non-classical monocytes (Supplementary Fig. 13). Within the overall decreasing dendritic cells, there was a relative increase in the proportion of plasmacytoid dendritic cells and a decrease in the proportion of myeloid and dendritic cells (Supplementary Fig. 14). The decrease in natural killer cell subpopulations is depicted in Supplementary Fig. 15. In the granulocytes, there was a significant increase in neutrophils after LPS infusion, while eosinophils and basophils were removed from circulation (Supplementary Fig. 16).

**Fig. 4:**
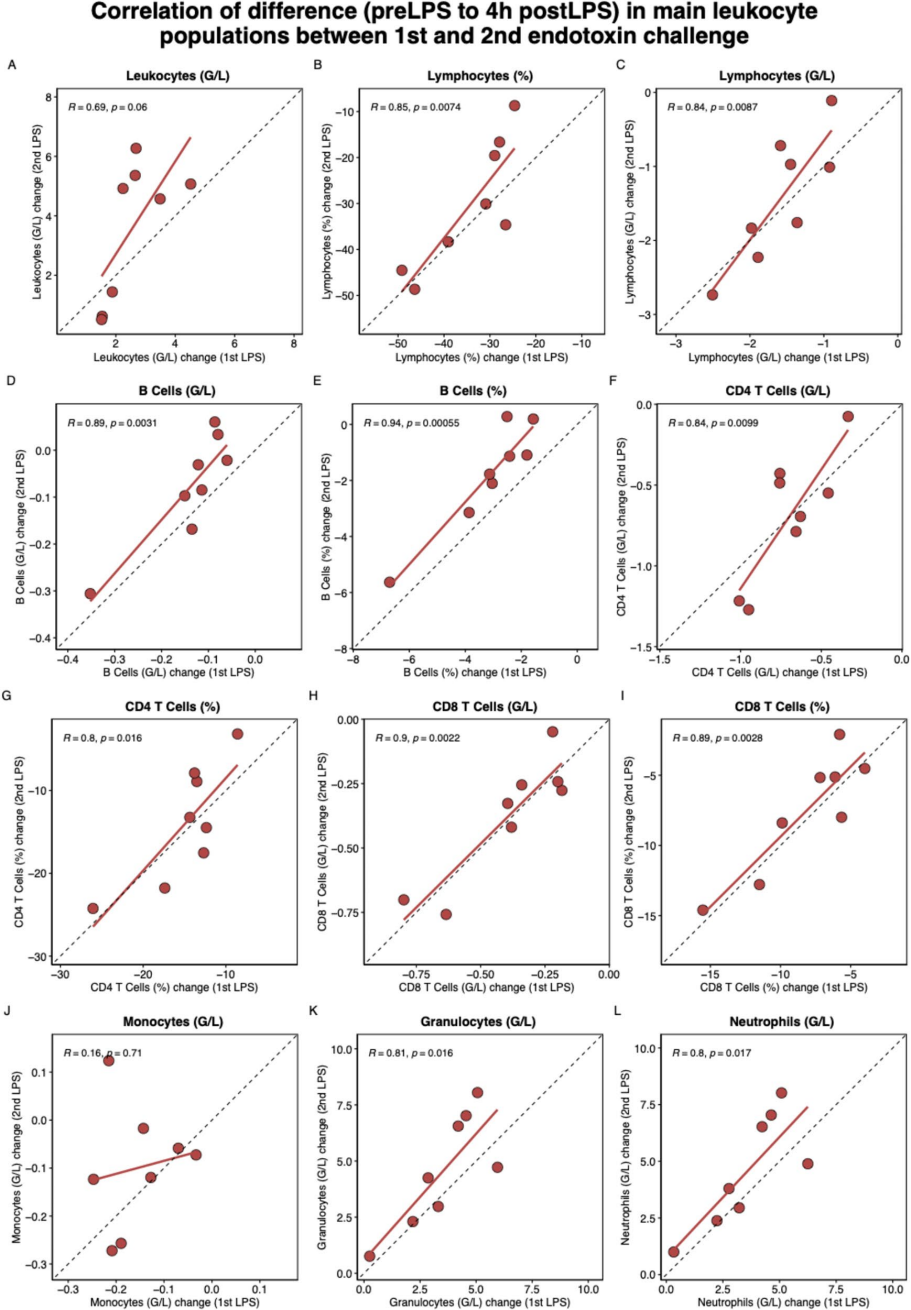
Correlation of difference (Δ baseline to 4 h) in main leukocyte populations between 1st and 2nd endotoxin challenge. Depicted are absolute (G/L) and relative (% of all leukocytes or % of the population) cell frequencies quantified by CyTOF. Because CyTOF quantification was unsuccessful in one female subject, only cell data of 8 of the 9 subjects. Each dot represents one participant. The dashed line indicates a perfect correlation. R is the Pearson correlation coefficient, accompanied by unadjusted p values

As shown in Fig. 4, a significant correlation in the cellular shift (Δ baseline to 4 h) was observed for absolute and relative lymphocytes, B cells, CD4 T cells, CD8 T cells, granulocytes, and neutrophils, but not for monocytes and the total leukocyte count. Supplementary Fig. 17 to 25 show the level of correlation of the changes in the respective subpopulations. Overall, the predominant proportion of subpopulations of B cells, CD4 and CD8 T cells, helper and regulatory T cells, natural killer T cells and gamma delta T cells, and dendritic cells showed a significant correlation between the 1st and the 2nd LPS challenge. Regarding monocytes, we found a significant correlation between transitional and non-classical, but not of classical monocyte subpopulations (Supplementary Fig. 22). In granulocytes, there was a significant correlation between the 1st and the 2nd LPS challenge for changes in neutrophils and basophils but not in eosinophils (Supplementary Fig. 25).

### Clinical response to LPS

**Fig. 5:**
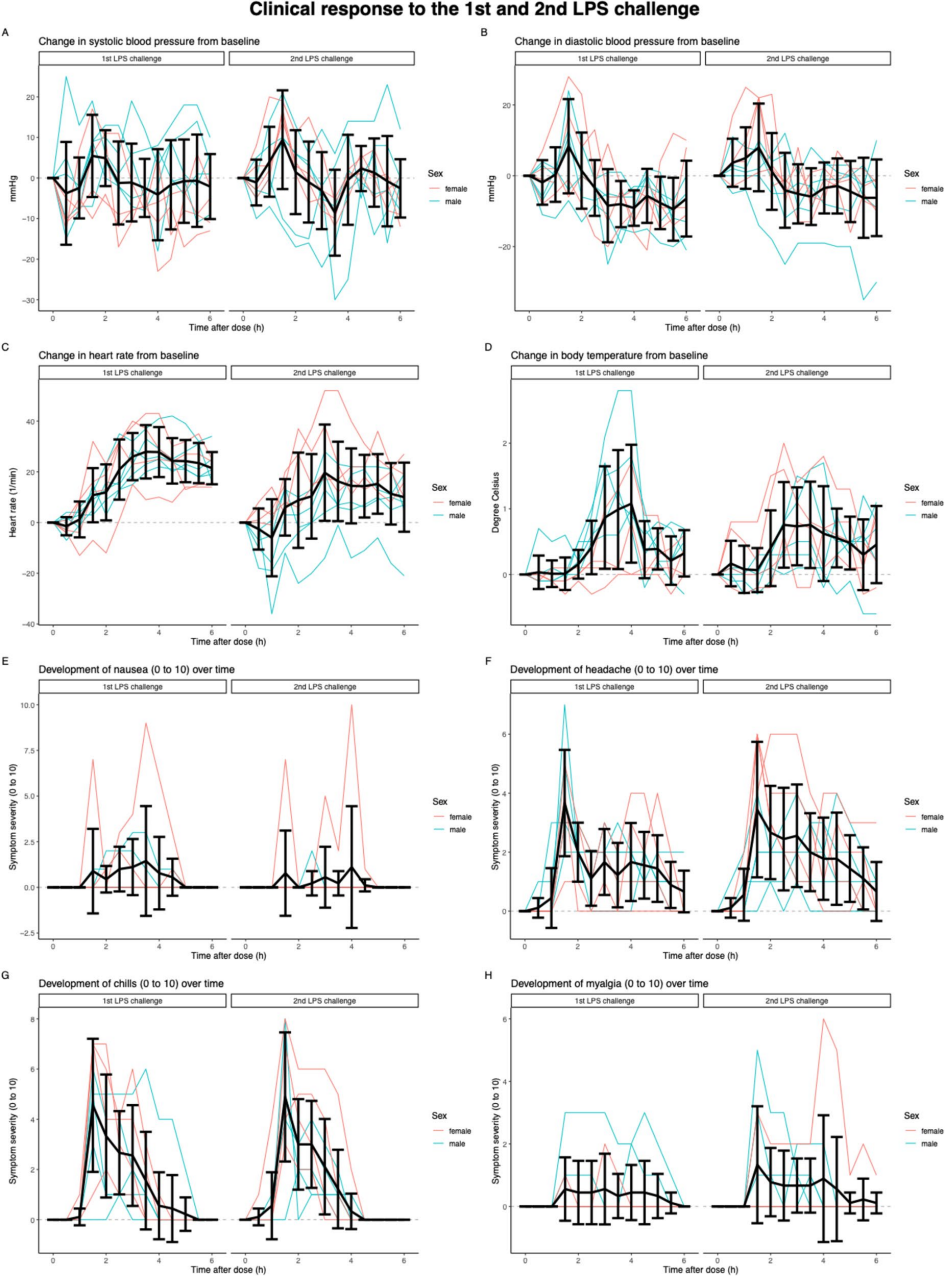
Clinical responses to 1st and 2nd LPS challenge. (**A**) systolic blood pressure (**B**) diastolic blood pressure (**C**) heart rate (**D**) body temperature (**E**) nausea (**F**) headache (**G**) chills (**H**) myalgia

**Fig. 6:**
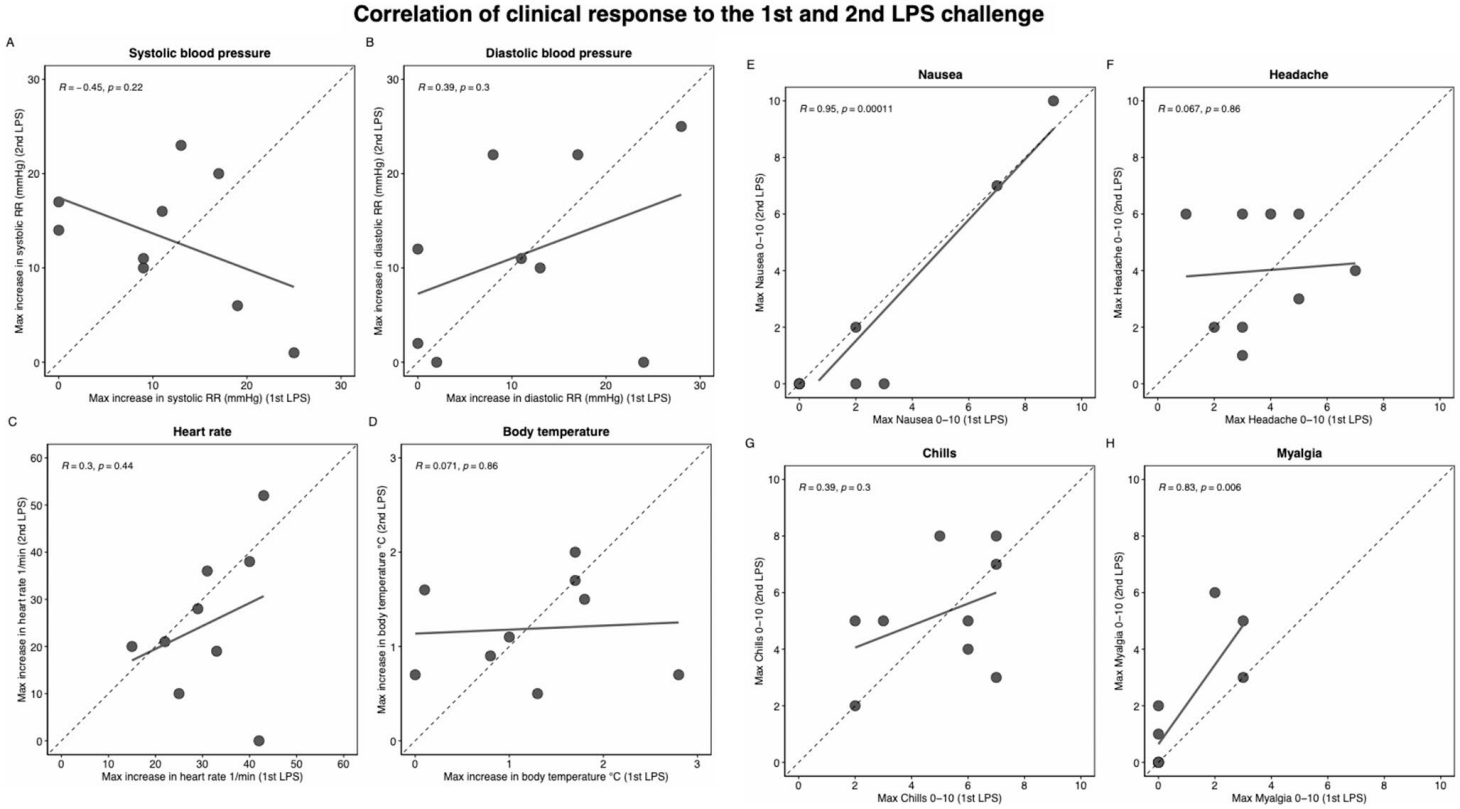
Correlation of clinical response to the 1st and 2nd LPS challenge (**A**) systolic blood pressure (**B**) diastolic blood pressure (**C**) heart rate (**D**) body temperature (**E**) nausea (**F**) headache (**G**) chills (**H**) myalgia

Figure 5 depicts the change in vital signs and severity of symptoms, which varied widely but did not differ significantly between the 1st and 2nd LPS challenges. Apart from the highest level of nausea (*R* = 0.95, *p* = 0.0001) and myalgia (*R* = 0.83, *p* = 0.006), no correlation between the change in vital signs and severity of symptoms after the 1st and 2nd LPS challenge was observed (Fig. 6). No serious adverse events occurred following the LPS infusion. Paracetamol administration was requested by one male (1 of 9 [11%]) subject in the 1st LPS challenge and by another male subject and four female (5 of 9 [56%])) subjects in the 2nd LPS challenge.

## Discussion

To the best of our knowledge, this is the first study in healthy volunteers to investigate the cytokine, cellular, and clinical response to repeated intravenous LPS challenges over a year apart. We report three main findings: First, we found that the biochemical (i.e., cytokines and CRP) and cellular responses showed statistically significant intra-individual correlation, which supports the reproducibility after repeated administration of LPS over an extended period of time. Second, and in contrast to the previous finding, clinical symptoms did not show consistent reproducibility. Third, the average magnitude of cytokine response, especially IL-6 and TNF-alpha, tended to be weaker after the 2nd LPS challenge, potentially implying a long-lasting endotoxin tolerance. The magnitude of the cellular response and clinical response was similar between the 1st and 2nd LPS challenge.

### Reproducibility of biochemical and cellular response to LPS

Several studies showed that repeated pulmonary LPS challenges produce consistent intra-subject cytokine and cellular responses after intervals of 4 to 5 weeks [14–16]. Similarly, in intravenous LPS challenges separated by at least 5 weeks, the extent of cytokine release within each subject was reproducible [6]. The present study indicates that IL-6, IL-8, TNF-alpha, and CRP show a strong intra-individual correlation after more than one year, a time span that has not been investigated so far.

Various previous studies explored the dynamic cellular changes in healthy volunteers following intravenous infusion of LPS, mostly focusing on one subset of leukocytes [17–19]. To our knowledge, this is the first study using a holistic immune profiling approach to describe the cellular dynamics after LPS infusion in healthy volunteers. Consistent with the available literature, our data showed that endotoxemia has a significant impact on virtually all circulating leukocyte populations, mainly characterized by an increase in neutrophils and a decrease in lymphocytes, monocytes, and other subsets of the innate immune system. Moreover, we observed that the extent of leukocyte subset changes after 4 h was stable within the individuals after the repeated LPS challenge, except for monocytes. We hypothesize that the recruitment and migration of leukocytes are significantly determined by individual constant factors, most likely (epi-)genetic factors or other constant factors such as sex or slowly changing factors such as age [20]. As previously described by Wegner et al. [21], females exhibited numerically greater AUCs and peak concentrations for all four cytokines during the 1st and 2nd LPS challenge, albeit without reaching statistical significance in the small sample size of this study (5 males vs. 4 females). Despite the lack of statistically significant sex differences, numerical differences may still have influenced the analyses presented in this work.

Monocyte dynamics did not show significant reproducibility, possibly because monocytes are highly plastic cells characterized by marked heterogeneity and versatility [22]. As a result, monocytes may be more influenced by environmental factors, compared with neutrophils or lymphocytes, which exhibited more stable long-term behavior and less pronounced turnover [22]. Since monocytes play an important role in the response to LPS, it could be hypothesized that the variability of the monocyte response could contribute to the variability of the clinical response.

The exact mechanism of cellular dynamics during endotoxemia is unknown, but the pronounced changes in virtually of subsets are likely caused by re-distribution. It has been shown that LPS administration increased endothelial activation markers including vascular cell adhesion molecule1 (VCAM1), thrombomodulin, and P-Selectin, which may result in the extravasation of particular subsets [23]. Cells may also be removed from the bloodstream by adhesion to and rolling on the endothelium and thus enter the so-called margination pool [24, 25], which is associated with systemic inflammation [26]. On the other hand, systemic inflammation induces the release of neutrophils from the bone marrow, which may help to explain the neutrophilia after LPS exposure [27, 28]. 

### Reproducibility of vital signs and symptoms after LPS infusion

Our study did not show a consistent reproducibility of the clinical response to intravenous LPS. The clinical manifestations are ultimately what inflammation research is interested in, as biochemical and cellular processes are merely biomarkers. Notably, clinical responses are difficult to quantify, partly subjective, and altogether more complex as they are the joint result of a multitude of determining factors. It is therefore not surprising that it is more challenging to show intra-individual reproducibility of the clinical response to LPS than that of biomarkers like cytokines. Moreover, the lack of reproducibility may indicate that clinical symptoms have – in addition to cytokine and cellular factors – even more sources of variability, such as environmental factors, day-to-day variability, or subjectiveness. In addition, 1 of 9 subjects received paracetamol in the 1st round, and 5 of 9 subjects received paracetamol in the 2nd round, despite a somewhat lower IL-6 response. Paracetamol was typically requested when symptom severity peaked. However, it is not known whether this prevented further worsening of symptoms and vital signs and thus introduced relevant confounding. We only observed a statistically significant correlation between the self-reported severity of nausea and myalgia. Whether these correlations are a product of chance (i.e., a type I error) or a slight indicator of intra-individual reproducibility of clinical responsiveness remains unclear and cannot be deduced from our data.

### Endotoxin tolerance

Numerous studies have demonstrated that repeated LPS exposure results in less pronounced cytokine responsiveness in healthy volunteers. However, the durability of this effect, which is commonly referred to as endotoxin tolerance, is undetermined [5, 29]. 

In a previous study at our site, we found lower levels of circulating cytokines following a repeated intravenous LPS challenge after 6 weeks [8]. Likewise, Janum et al. also found a lower cytokine release following a second intravenous LPS challenge in a crossover trial with a washout period of 4 weeks [30]. 

In the present study, we found a significantly reduced release of IL-6 and TNF-alpha after the 2nd LPS challenge, possibly indicating long-lasting endotoxin tolerance. IL-10, IL-8, and CRP were also numerically lower after the 2nd LPS challenge, but without becoming statistically significant. The kinetic profile, which was characterized by peak concentrations at around 2 h followed by a clearance until 6 to 10 h after LPS administration, remained similar during the repeated endotoxemia. Regarding the cellular response, we found few statistically significant signals indicating a lower or higher responsiveness at the 2nd LPS challenge. Given the large number of comparisons of relative and absolute cellular subsets and the lack of a consistent and plausible pattern in these differences, we do not believe that our data robustly supports an attenuated cellular response after repeated LPS infusion.

Although these results should be interpreted with caution, we cannot rule out some degree of endotoxin tolerance, which may be associated with an attenuated cytokine response, even after an extended period of time. As Netea et al. pointed out, current evidence suggests that the reprogramming of the innate immune system can last for periods of time on the order of years [3, 29, 31]. While the exact mechanisms of immune tolerance are still being explored, the important role of epigenetic reprogramming, which could also help explain the long-lasting effect that extends well beyond the lifespan of circulating mature leukocytes, is increasingly recognized [3, 32]. Subsequently, responsiveness to LPS may also be influenced by previous bacterial infections.

LPS is considered as a potential adjuvant for vaccines, but its unmodified form is limited by its toxicity. LPS has therefore been detoxified to monophosphorylated lipid A (MPL) [33] to stimulate TLR4-mediated responses while reducing systemic inflammatory toxicity. For example, MPL is currently used in the licensed shingles vaccine Shingrix^®^. On the other hand, meningococcal vaccines use outer membrane vesicles containing lipooligosaccharides – the Neisseria meningitidis analogue of LPS that lacks the repeated residues of the O antigen [34] – which are also chemically detoxified [35]. In contrast to the intravenous LPS challenge, the LPS components in these vaccines are designed to stimulate the adaptive immune response without inducing pronounced systemic inflammation. Given the observed tolerance to repeated intravenous administration of LPS, it is conceivable that this tolerance could also be found to modified LPS or lipid A, potentially impairing immunogenicity after vaccination. Considering the modest degree of LPS tolerance observed in our study, it is unlikely to play a clinically relevant role by reducing immune responses or even protection after extended time periods. Since TLR4 signaling and subsequent cytokine responses depend on the modifications of LPS [36], strain-specific differences in the spectrum of endotoxicity should also be considered when interpretating these data [37]. 

### Limitations

First, this was an exploratory study in which no adjustment for multiple comparisons was made. Because of the large number of statistical tests, our results are at considerable risk of false positivity and should be interpreted as hypothesis-generating only. Second, quantification of cellular subsets using CyTOF was performed at 0 and 4 h, which only provides an incomplete temporal resolution of the complex cellular dynamics following endotoxemia. Third, the number of subjects was small (9 for the cytokine and clinical response and 8 for the cellular response). However, establishing statistically significant correlations with such a small sample size is only possible in the case of strong and thus potentially meaningful correlations.

Fourth, seasonality is another important source of variability [20], for which controlling was not feasible for us. Notably, technical and study design-related sources of variability would bias toward the null hypothesis and against the finding of a statistically significant correlation. Finally, we acknowledge that we studied statistical differences within a model for systemic inflammation in young healthy volunteers. While our findings may help to hypothesize immunological mechanisms, the clinical relevance of the observed results is unknown without confirmatory studies.

## Conclusions

This study demonstrated that the cytokine and cellular response to intravenous LPS exhibits a significant degree of intraindividual reproducibility, even when performed more than one year apart. These correlations did not translate to the reproducibility of clinical symptoms and vital signs, which showed greater variability and were not constant over time. While the average magnitude of cellular and clinical response was similar between the two LPS exposures, we found some evidence of attenuated cytokine release after the 2nd LPS exposure, possibly indicating long-lasting residual tolerance to endotoxin. In summary, our results suggest that the individual responsiveness of the innate immune system is maintained over prolonged periods, indicating that – in addition to rapidly changing environmental factors – enduring host factors, such as epigenetic or genetic features, determine our biochemical and cellular immune response to stimuli. These findings should encourage future studies aimed at identifying host-specific factors that determine the magnitude of the human innate immune response and that could be explored as potential treatment targets or prognostic markers.

## Electronic supplementary material

Below is the link to the electronic supplementary material.

Supplementary Material 1

## Data Availability

No datasets were generated or analysed during the current study.
