# Supplementary material for: Stability of cytokine, cellular and clinical response to the intravenous LPS challenge repeated after one year: a healthy volunteer trial
Source: Med Microbiol Immunol. 2025 Mar 6;214(1):14. doi: 10.1007/s00430-025-00823-5 (PMC11885351; doi:10.1007/s00430-025-00823-5)
Supplement: Supplementary file 1 — Supplementary Material 1 [file 430_2025_823_MOESM1_ESM.pdf]

Supplementary Appendix to:

Stability of cytokine, cellular and clinical response to the intravenous LPS challenge repeated after one year: a healthy volunteer trial

Tables

Supplementary Table 1 Inclusion and exclusion criteria

**Inclusion criteria**
• Written informed consent was given by volunteers after being provided detailed information about the nature, risks, and scope of the study.<br>• Healthy according to the medical history, ECG, vital signs, laboratory results, and physical examination<br>• Male and female participants<br>• Age: ≥20 to ≤30 years<br>• For women of childbearing age: use of effective birth control or abstinence from heterosexual intercourse with fertile men
**Exclusion criteria**
• Known or suspected allergy to LPS<br>• History of severe allergic or anaphylactic reactions to any medication<br>• Pregnancy or no current birth control method (woman of childbearing age)<br>• Blood or plasma donation within the last 4 weeks before the scheduled day of the first blood draw or anytime during the study<br>• Treatment with an investigational drug within three weeks before the study day<br>• Smoking more than 5 cigarettes per day<br>• History of substance abuse<br>• Regular use of medication other than birth control<br>• Use of any medication within one week before study days except birth control<br>• Symptoms of a clinically relevant illness in the 3 months before the study day<br>• Known coagulation disorder, i.e., factor V Leiden mutation.<br>• Liver or kidney dysfunction<br>• Positive serology for hepatitis or HIV<br>• Weight >95 kg and ≤60 kg for men or ≤50 kg for women<br>• Other objections to participating in the study in the opinion of the investigator.<br>• Any vaccination (including but not limited to COVID-19 and Influenza vaccines) within 4 weeks before study day 1<br>• Previous participation in an intravenous LPS study

Supplementary Table 2 List of lifestyle restrictions before the study days

**Restrictions**
• Subjects will have to refrain from any medication (other than birth control) one week before study days.<br>• Subjects will have to refrain from alcohol, caffeine-containing beverages, or energy drinks 6 hours before study days.<br>• Subjects will have to ensure a sleep duration of at least 7 hours and a fasting period of at least 8 hours before study days.<br>• Subjects will be not allowed to receive any kind of vaccination (including but not limited to COVID-19 and Influenza vaccines) 4 weeks before the first study days until the last study day.

Supplementary Table 3 Performance of repeatedly measured quality control (QC) samples

**Low Concentration**	**IL-8 (pg/mL)**	**IL-10 (pg/mL)**	**IL-6 (pg/mL)**	**TNFa (pg/mL)**
QC_low Plate 1	29.1	702.1	153.4	141.3
QC_low Plate 2	24.7	476.0	132.6	119.3
QC_low Plate 3	25.3	589.1	130.9	111.0
QC_low Plate 4	22.8	572.2	117.7	97.3
QC_low Plate 5	30.9	586.5	97.5	122.9
Standard deviation	3.0	71.8	18.4	14.4
Mean	26.6	585.2	126.4	118.4
CoV %	11.3	12.3	14.6	12.2
**High Concentration**	**IL-8 (pg/mL)**	**IL-10 (pg/mL)**	**IL-6 (pg/mL)**	**TNFa (pg/mL)**
QC_high Plate 1	224.3	5754.5	1215.2	1170.8
QC_high Plate 2	220.5	5664.4	1075.2	1153.1
QC_high Plate 3	184.3	5935.0	1029.8	1131.3
QC_high Plate 4	224.8	5431.7	1203.4	1163.9
QC_high Plate 5	439.1	8510.2	1439.0	1690.7
Standard deviation	91.5	1137.1	142.6	214.8
Mean	258.6	6259.2	1192.5	1262.0
CoV %	35.4	18.2	12.0	17.0

Supplementary Table 4 Individual AUCs$_{0-10}$ of cytokine responses and 24-h concentration of C-reactive protein after the 1st and 2nd endotoxin challenge

Index	Population	Markers
1	Lymphocytes	CD3 T cells + B cells + NK cells + plasma b cells
2	CD3 T cells	CD8 T cells + CD4 T cells + à T cells + MAIT/NKT cells
3	CD8 T cells	CD3+ CD66b- CD19- CD8+ CD4- CD14- CD161- TCRgd- CD123- CD11c-
4	CD8 naïve	CD8 T cells + CD45RA+ CCR7+ CD27+
5	CD8 central memory	CD8 T cells + CD45RA- CCR7+ CD27+
6	CD8 effector memory	CD8 T cells + CCR7- CD27+
7	CD8 terminal effector	CD8 T cells + CCR7- CD27-
8	CD4 T cells	CD66b- CD3+ CD8- CD4+ CD14- TCRgd- CD11c-
9	CD4 naïve	CD4 T cells + CD45RA+ CCR7+ CD27+
10	CD4 central memory	CD4 T cells + CD45RA- CCR7+ CD27+
11	CD4 effector memory	CD4 T cells + CD45RA- CCR7- CD27+
12	CD4 terminal effector	CD4 T cells + CD45RA- CCR7- CD27-
13	Tregs	CD4 T cells + CD25+ CD127- CCR4+
14	Th1-like	CD4 T cells + CXCR3+ CCR6- CXCR5- CCR4-
15	Th2-like	CD4 T cells + CXCR3- CCR6- CXCR5- CCR4+
16	Th17-like	CD4 T cells + CXCR3- CCR6+ CXCR5- CCR4+
17	T cells	CD66b- CD3+ CD8dim,- CD4- CD14- TCRgd dim,+
18	NKT cells	CD66b- CD3+ CD4- CD14- CD161+ TCRgd- CD28+ CD16-
19	B cells	CD3- CD14- CD56- CD16 dim,- CD19+ CD20+ HLA-DR dim,+
20	B naïve	B cells + CD27-
21	B memory	B cells + CD27+
22	Plasma b cells	CD3- CD14- CD16-,dim CD66b- CD20- CD19+ CD56- CD38++ CD27+
23	NK cells	CD14- CD3- CD123- CD66b- CD45RA+ CD56 dim,+
24	NK early	NK cells + CD57-
25	NK late	NK cells + CD57+
26	Monocytes	CD3- CD19- CD56- CD66b- HLA-DR+ CD11c+
27	Monocytes classical	Monocytes + CD14+ CD38+
28	Monocytes transitional	Monocytes + CD14 dim CD38 dim
29	Monocytes non-classical	Monocytes + CD14- CD38-
30	DCs	pDCs + mDCs
31	pDCs	CD3- CD19- CD14- CD20- CD66b- HLA-DR dim,+ CD11c- CD123+
32	mDCs	CD3- CD19- CD14- CD20- HLA-DR dim,+ CD11c dim,+ CD123- CD16 dim,- CD38 dim,+ CD294- HLA-D
33	Granulocytes	Neutrophils + basophils + eosinophils + CD66b- neutrophils
34	Neutrophils	CD66b dim,+ CD16+ HLA-DR-
35	Basophils	HLA-DR- CD66b- CD123 dim,+ CD38+ CD294+
36	Eosinophils	CD14- CD3- CD19- HLA-DR- CD294+ CD66b dim,+

Supplementary Table 5 Individual $AUCs_{0-10}$ of cytokine responses and 24-h concentration of C-reactive protein after the 1st and 2nd endotoxin challenge

	**Overall**	**1st LPS challenge**	**2nd LPS challenge**
**N=**	9 subjects twice	9	9
Interleukin 8, AUC (pg*h/mL), (mean [SD])	883 (606)	1047 (583)	718 (616)
Interleukin 8, Cmax (pg/mL), (mean [SD])	385 (250)	434 (234)	336 (269)
Interleukin 10, AUC (pg*h/mL), (mean [SD])	1771 (802)	1941 (753)	1600 (856)
Interleukin 10, Cmax (pg/mL), (mean [SD])	535 (300)	582 (331)	487 (277)
Interleukin 6, AUC (pg*h/mL), (mean [SD])	2150 (2830)	2719 (2798)	1581 (2909)
Interleukin 6, Cmax (pg/mL), (mean [SD])	868 (844)	1114 (761)	623 (895)
TNF alpha, AUC (pg*h/mL), (mean [SD])	2019 (1365)	2459 (1191)	1579 (1450)
TNF alpha, Cmax (pg/mL), (mean [SD])	704 (502)	859 (426)	549 (548)
C-reactive protein, 24h Conc (mg/dL), (mean [SD])	3.0 (1.6)	3.2 (1.3)	2.8 (1.8)

Supplementary Table 6 Analyses of variance (ANOVAs) testing the association of the LPS challenge (1st vs 2nd) and the interaction between the LPS challenge (1st vs 2nd) and the concentration per timepoint for each cytokine

	Effect of challenge (1st vs 2nd)			Interaction challenge:timepoint		
	Degree of freedom	F value	p value	Degree of freedom	F value	p value
Interleukin-8	1	7.027	0.23	7	2.365	0.0365
Interleukin-10	1	0.349	0.66	7	0.789	0.6
Interleukin-6	1	13.38	0.17	7	1.749	0.119
TNF-alpha	1	842.8	0.0219	7	4.086	0.00133

Supplementary Table 7 Comparisons of peak concentrations (C_{max}), time to reach peak concentrations (T_{max}), and areas under the curve (AUC) between the 1st and 2nd LPS challenge for (A) interleukin-8, (B) interleukin-10, (C) interleukin-6, and (D) tumor necrosis factor-alpha.

	**Parameter**	**1st Challenge, Mean (SD)**	**2nd Challenge, Mean (SD)**	**Mean Difference (95% CI)**	**P value**
**Interleukin-8**	C_{max} (pg/mL)	434.13 (234.06)	335.73 (269.27)	-98.40 (-292.41, 95.62)	0.276
	T_{max} (h)	2.22 (0.67)	2.00 (0.00)	-0.22 (-0.73, 0.29)	0.347
	AUC_{0-10} (pg/mL*h)	1047.41 (582.68)	718.13 (615.53)	-329.28 (-679.04, 20.48)	0.062
**Interleukin-10**	C_{max} (pg/mL)	582.39 (330.79)	487.43 (277.11)	-94.97 (-370.83, 180.90)	0.450
	T_{max} (h)	2.44 (0.88)	2.39 (0.93)	-0.06 (-0.83, 0.72)	0.873
	AUC_{0-10} (pg/mL*h)	1941.08 (753.13)	1600.01 (856.03)	-341.07 (-1011.12, 328.98)	0.274
**Interleukin-6**	C_{max} (pg/mL)	1113.84 (760.89)	622.87 (894.90)	-490.96 (-877.88, -104.04)	0.019
	T_{max} (h)	2.22 (0.67)	2.22 (0.67)	-0.00 (NA, NA)	NA
	AUC_{0-10} (pg/mL*h)	2719.23 (2797.83)	1581.12 (2908.53)	-1138.11 (-1985.01, -291.20)	0.015
**TNF-alpha**	C_{max} (pg/mL)	858.79 (425.50)	549.42 (548.29)	-309.38 (-621.37, 2.62)	0.052
	T_{max} (h)	1.78 (0.26)	1.56 (0.30)	-0.22 (-0.56, 0.12)	0.169
	AUC_{0-10} (pg/mL*h)	2459.34 (1191.47)	1579.38 (1450.09)	-879.96 (-1719.66, -40.26)	0.042

Note: The statistical comparison is based on the paired-sample t-test.

Supplementary Table 8 Comparisons of peak concentrations (C_{max}), time to reach peak concentrations (T_{max}), and areas under the curve (AUC) between male and female subjects during the 1st and 2nd LPS challenge.

**1st LPS Challenge**					
	Parameter	Males, Mean (SD) N=5	Females, Mean (SD) N=4	Mean difference (95% CI)	P value
**Interleukin-8**	C_{max} (pg/mL)	400 (274)	476 (204)	76 (-301, 453)	0.649
	T_{max} (h)	2.4 (0.9)	2.0 (0.0)	-0.4 (-1.5, 0.7)	0.407
	AUC_{0-10} (pg/mL*h)	960 (500)	1157 (737)	197 (-904, 1299)	0.666
**Interleukin-10**	C_{max} (pg/mL)	453 (140)	744 (451)	290 (-401, 981)	0.292
	T_{max} (h)	2.8 (1.1)	2.0 (0.0)	-0.8 (-2.1, 0.5)	0.193
	AUC_{0-10} (pg/mL*h)	1611 (422)	2353 (932)	741.9 (-656, 2139)	0.214
**Interleukin-6**	C_{max} (pg/mL)	757 (428)	986 (447)	230 (-479, 938)	0.463
	T_{max} (h)	1.7 (0.3)	1.9 (0.3)	0.2 (-0.2, 0.6)	0.351
	AUC_{0-10} (pg/mL*h)	2160 (1157)	2833 (1290)	673 (-1337, 2682)	0.446
**TNF-alpha**	C_{max} (pg/mL)	938 (434)	1333 (1085)	395 (-1244, 2033)	0.533
	T_{max} (h)	2.0 (0.0)	2.5 (1.0)	0.5 (-0.5, 1.5)	0.292
	AUC_{0-10} (pg/mL*h)	2101 (1041)	3491 (4242)	1390 (-5189, 7969)	0.564
**2nd LPS Challenge**					
		Males, Mean (SD)	Females, Mean (SD)	Mean difference (95% CI)	P value
**Interleukin-8**	C_{max} (pg/mL)	230 (93)	468 (374)	238 (-341, 817)	0.295
	T_{max} (h)	2.0 (0.0)	2.0 (0.0)	0.0 (NA,NA)	NA
	AUC_{0-10} (pg/mL*h)	457 (117)	1045 (858)	588 (-765, 1941)	0.264
**Interleukin-10**	C_{max} (pg/mL)	442 (321)	544 (244)	101 (-344, 547)	0.607
	T_{max} (h)	2.3 (1.0)	2.5 (1.0)	0.2 (-1.4, 1.8)	0.772
	AUC_{0-10} (pg/mL*h)	1562 (1014)	1647 (760)	85 (-1314, 1485)	0.890
**Interleukin-6**	C_{max} (pg/mL)	274 (81)	894 (713)	620 (-507, 1747)	0.180
	T_{max} (h)	1.50 (0.4)	1.6 (0.3)	0.1 (-0.4, 0.6)	0.555
	AUC_{0-10} (pg/mL*h)	826 (198)	2521 (1851)	1695 (-1234, 4624)	0.164
**TNF-alpha**	C_{max} (pg/mL)	175 (73)	1183 (1173)	1008 (-855, 2871)	0.184
	T_{max} (h)	2.0 (0.0)	2.5 (1.0)	0.5 (-0.5, 1.5)	0.292
	AUC_{0-10} (pg/mL*h)	377 (155)	3087 (4133)	2710 (-3862, 9283)	0.281

Note: The statistical comparison is based on the independent-sample t-test.

Figures

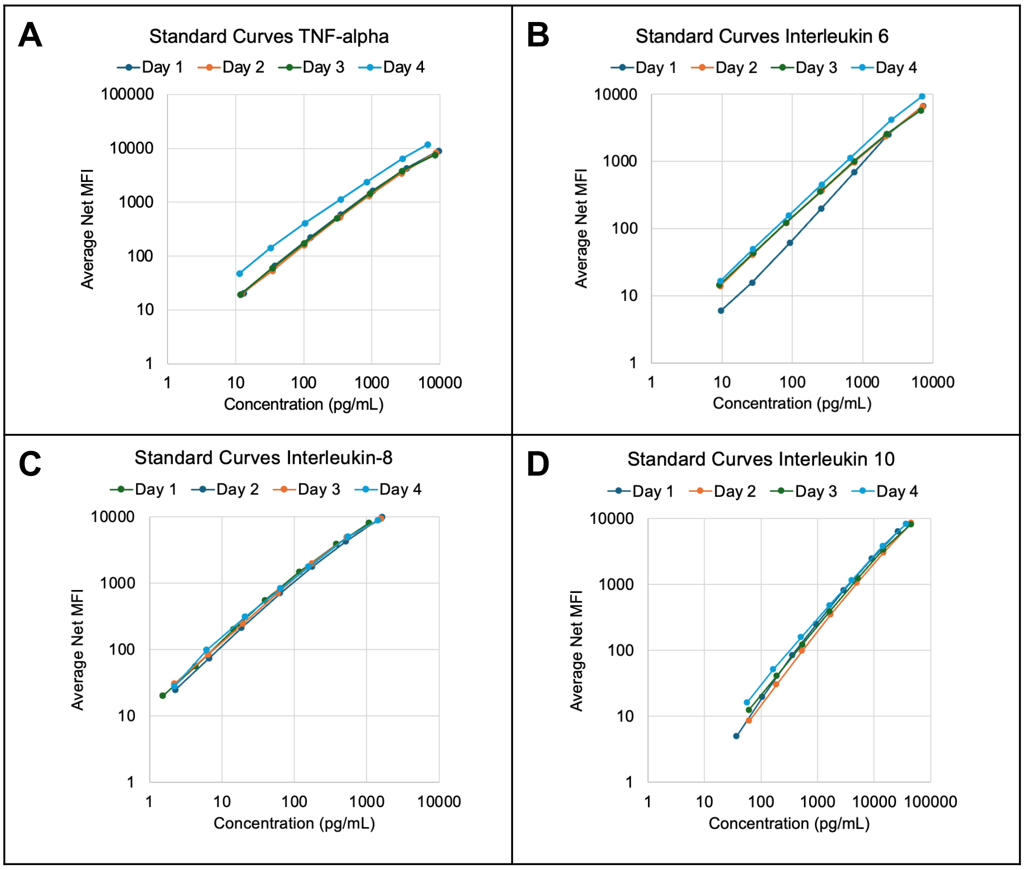

Supplementary Figure 1 Standard curves for (A) Tumor necrosis-alpha, (B) interleukin-6, (C)interleukin-8, and (D) interleukin-10. The standard curves were created on four different measurement days

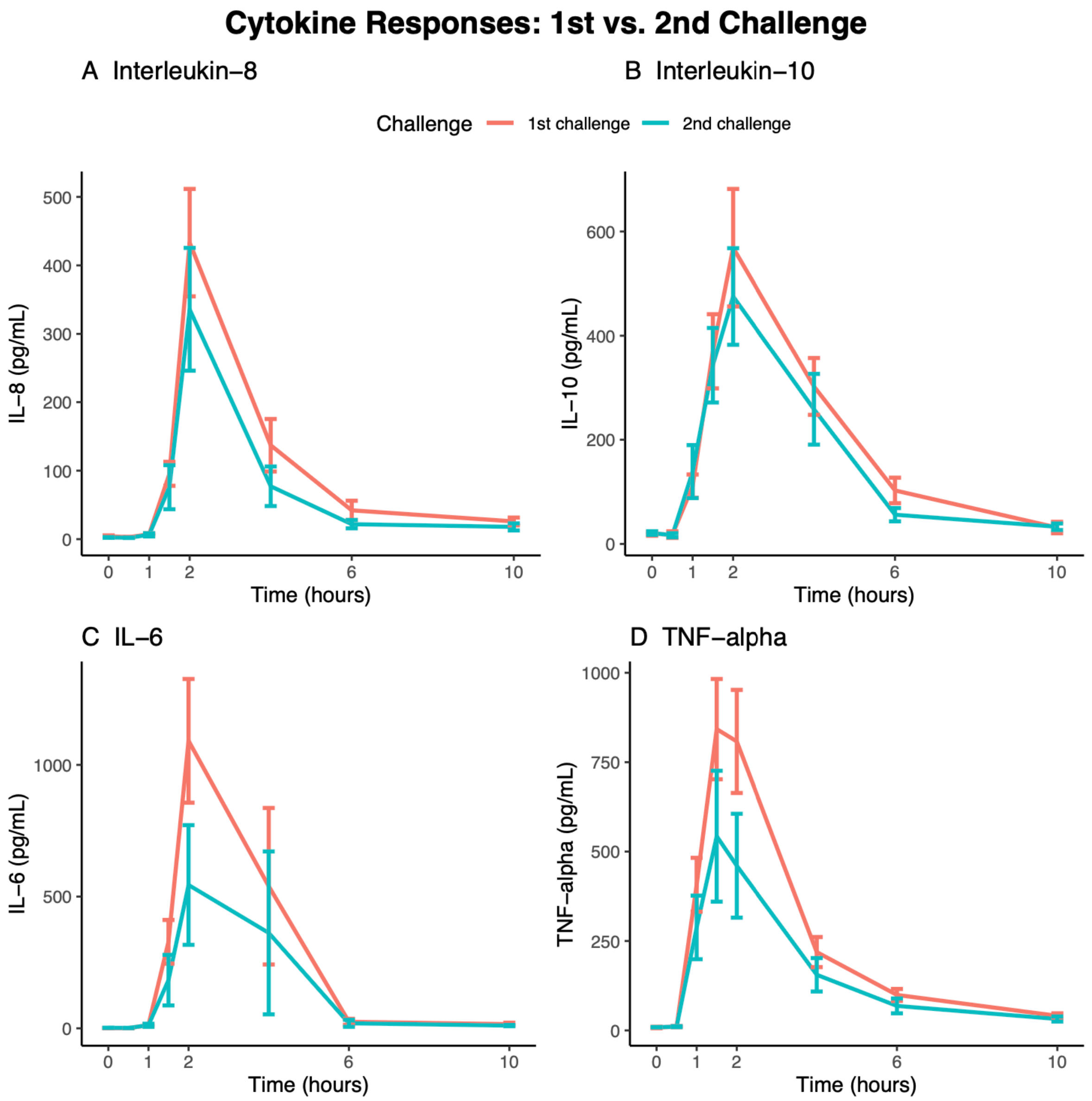

Supplementary Figure 2 Individual concentration-time profiles between the 1st (red line) and 2nd (green line) LPS challenge for (A) interleukin-8, (B) interleukin-10, (C) interleukin-6, and (D) tumor necrosis factor-alpha. Error bars indicate the standard error.

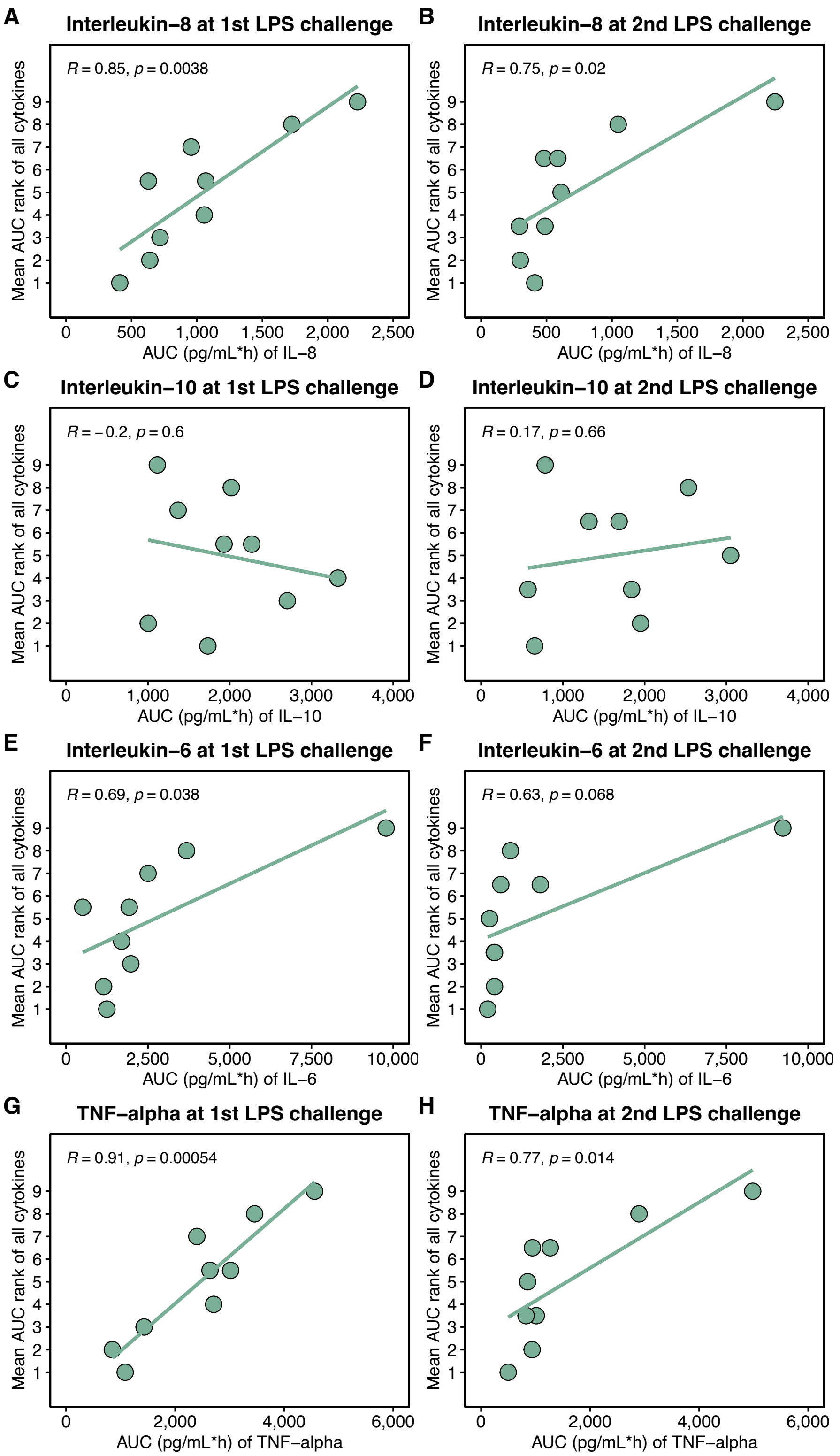

Supplementary Figure 3 Dot plots assessing the correlation between the individual cytokine $AUCs_{0\text{-}10}$ and the mean AUC rank during the 1st and 2nd LPS challenge. (A) interleukin-8 at 1st LPS challenge, (B) interleukin-8 at 2nd LPS challenge, (C) interleukin-10 at 1st LPS challenge, (D) interleukin-10 at 2nd LPS challenge, (E) interleukin-6 at 1st LPS challenge, (F) interleukin-6 at 2nd LPS challenge, (G) TNF-alpha at 1st LPS challenge, (G) TNF-alpha at 2nd LPS challenge.

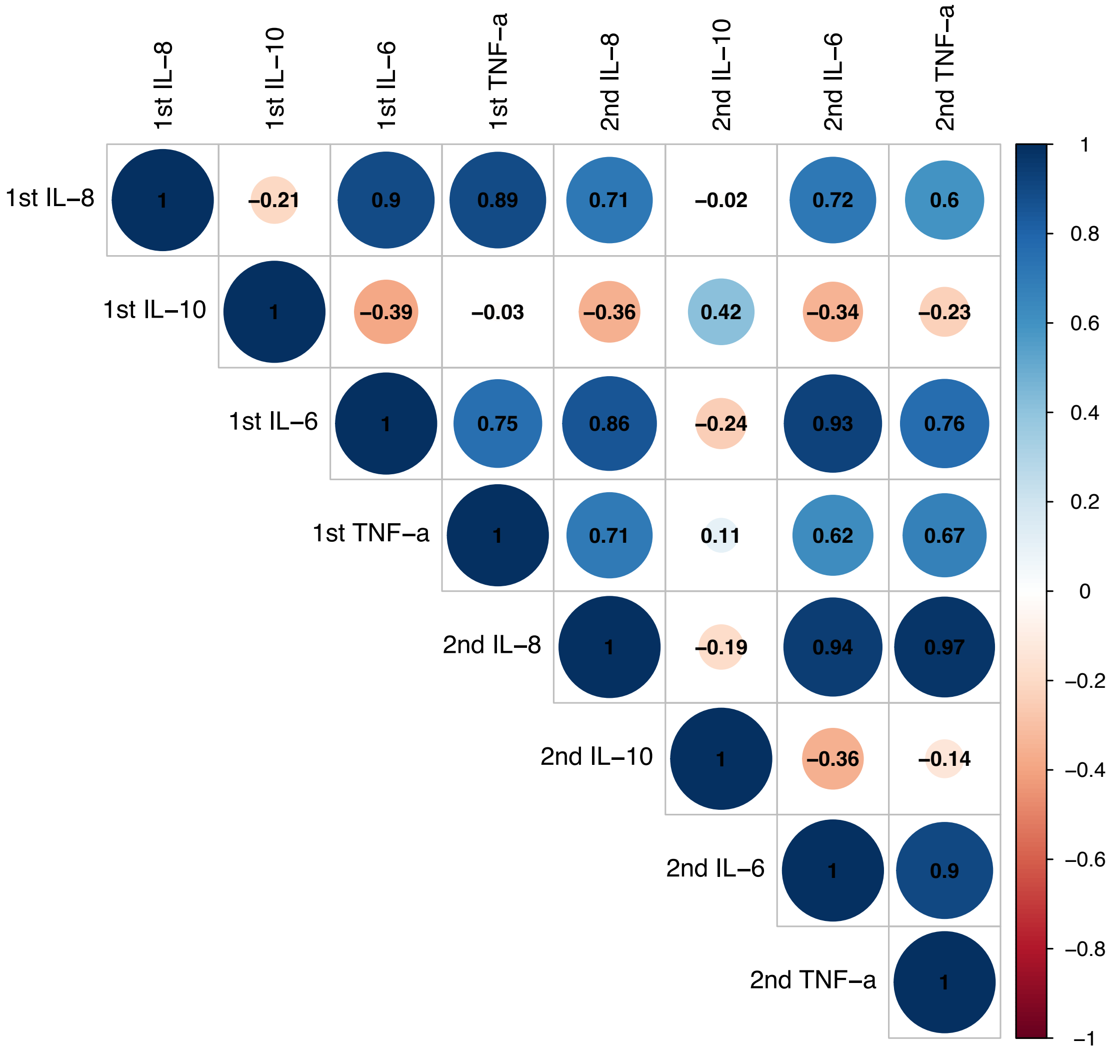

Supplementary Figure 4 Correlation plot of individual cytokine AUCs$_{0\text{-}10}$ during the 1st and 2nd LPS challenge. The values and the size of the circle indicate the degree of correlation, expressed by the Pearson correlation coefficient R.

Difference in B cell subsets changes between the 1st and 2nd LPS challenge

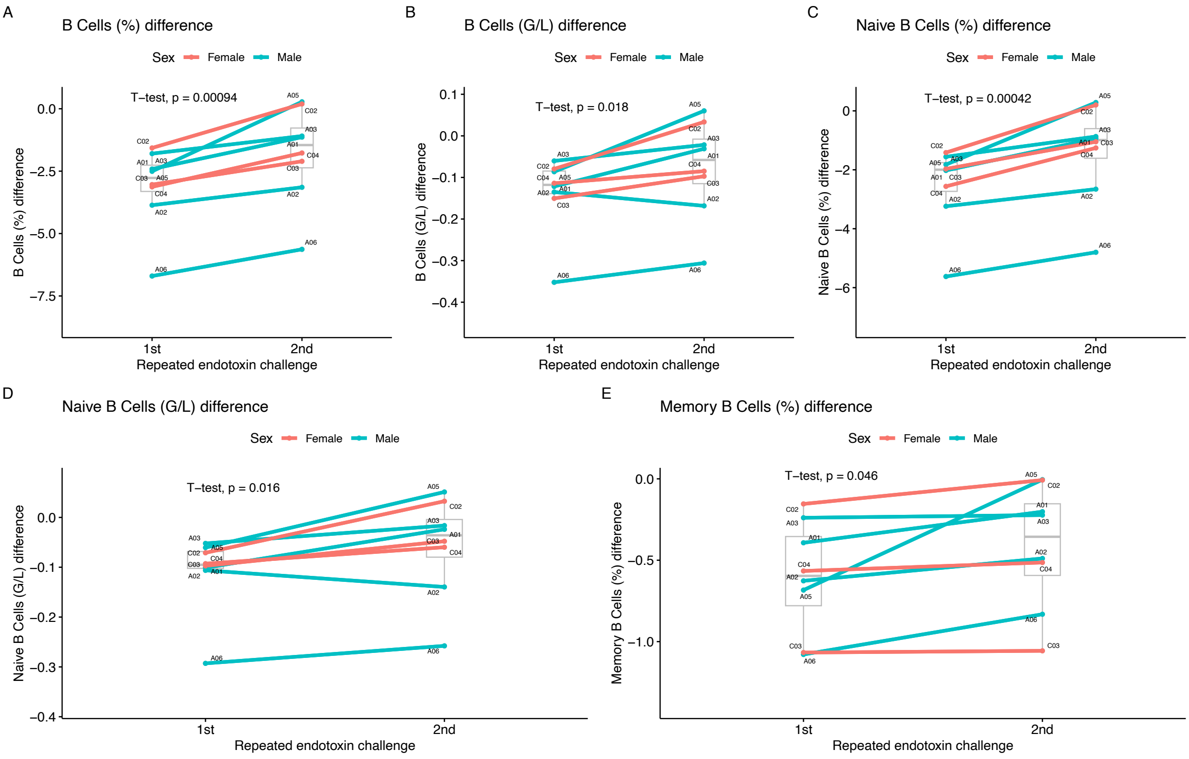

Supplementary Figure 5 Selection of statistically significant changes (Δ baseline to 4h) in B lymphocyte subsets between the 1st and 2nd LPS challenge

Difference in T cell subsets changes between the 1st and 2nd LPS challenge

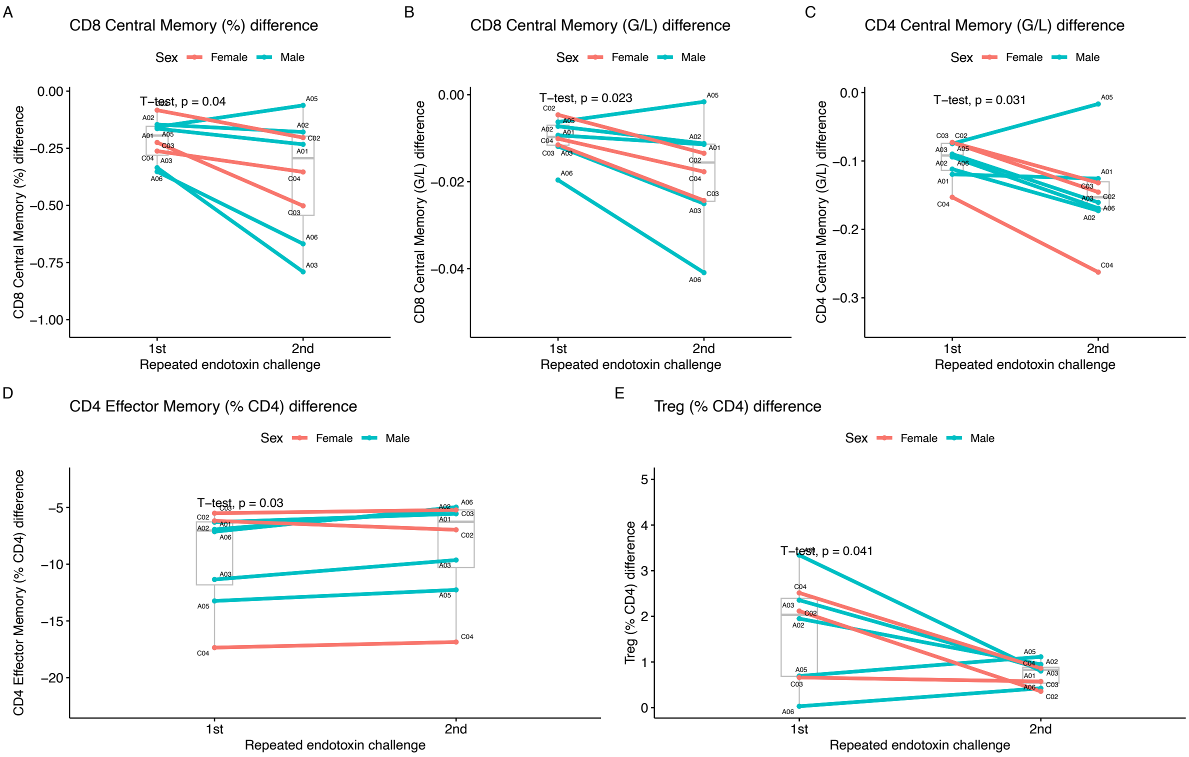

Supplementary Figure 6 Selection of statistically significant changes (∆ baseline to 4h) in T lymphocyte subsets between the 1st and 2nd LPS challenge

Difference in dendritc cell subsets and basophils changes between the 1st and 2nd LPS challenge

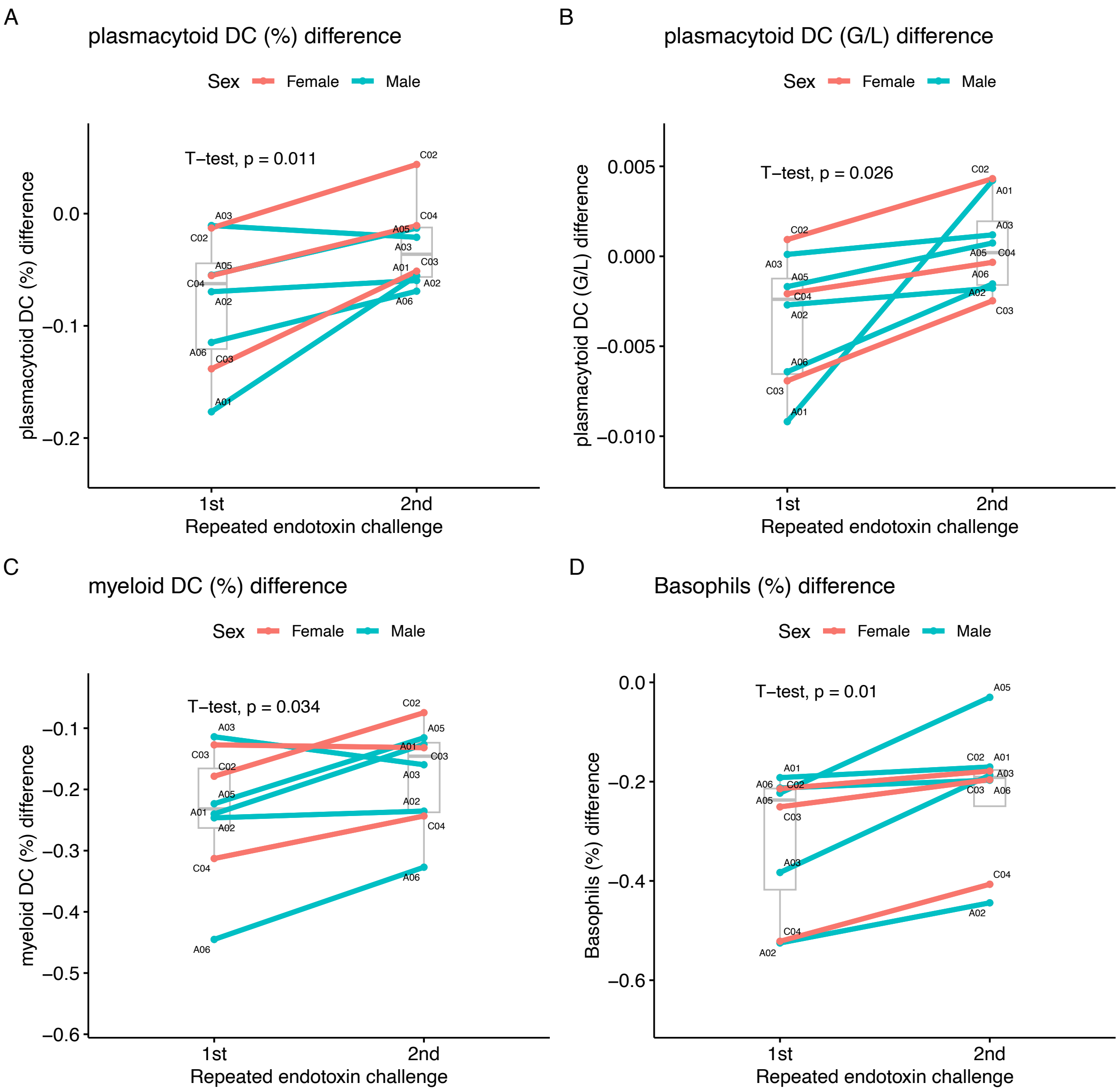

Supplementary Figure 7 Selection of statistically significant changes (Δ baseline to 4h) in dendritic cells subsets and basophils between the 1st and 2nd LPS challenge

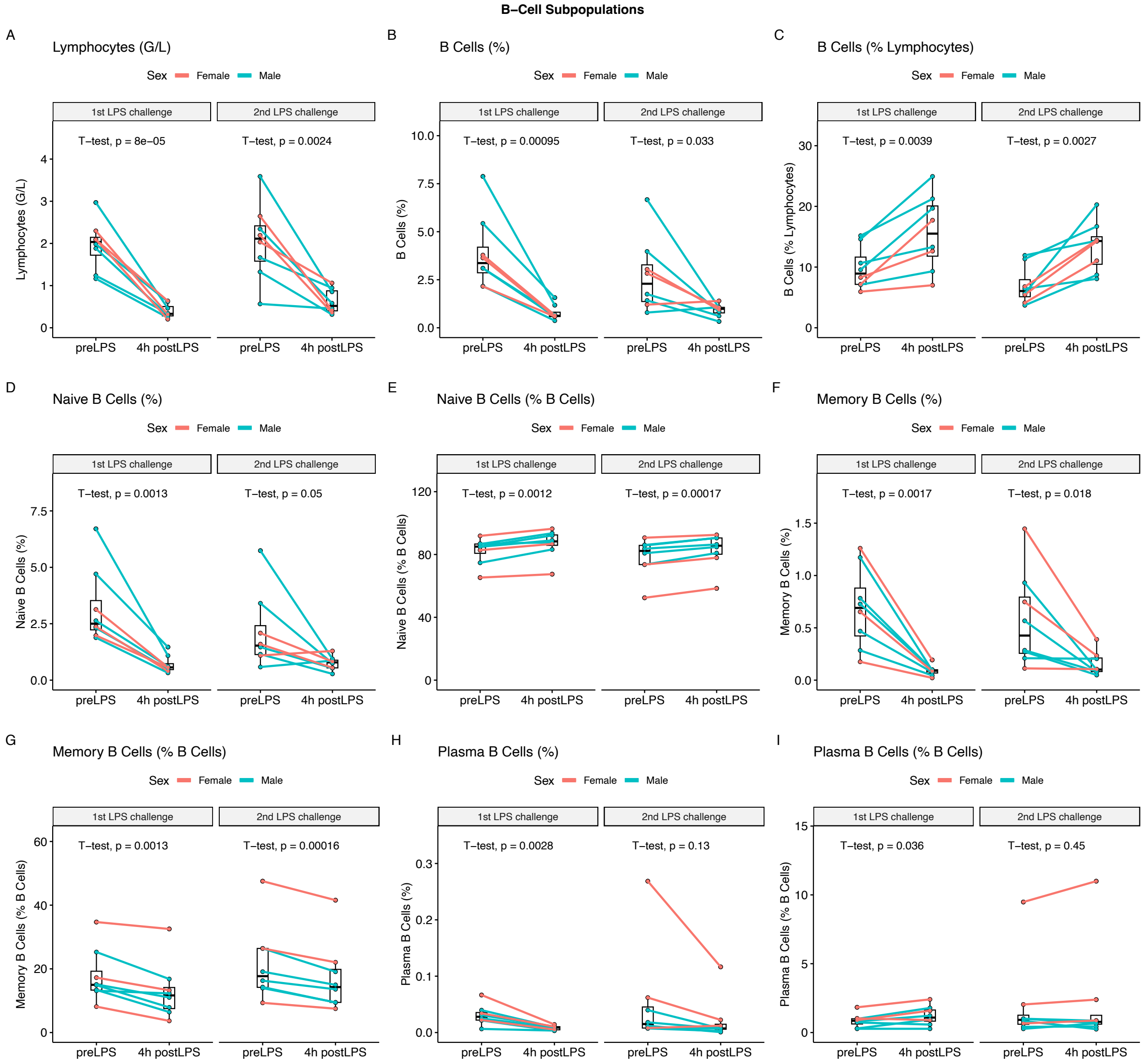

Supplementary Figure 8 B lymphocyte subpopulations before and 4h after LPS administration at the 1st and 2nd LPS challenge

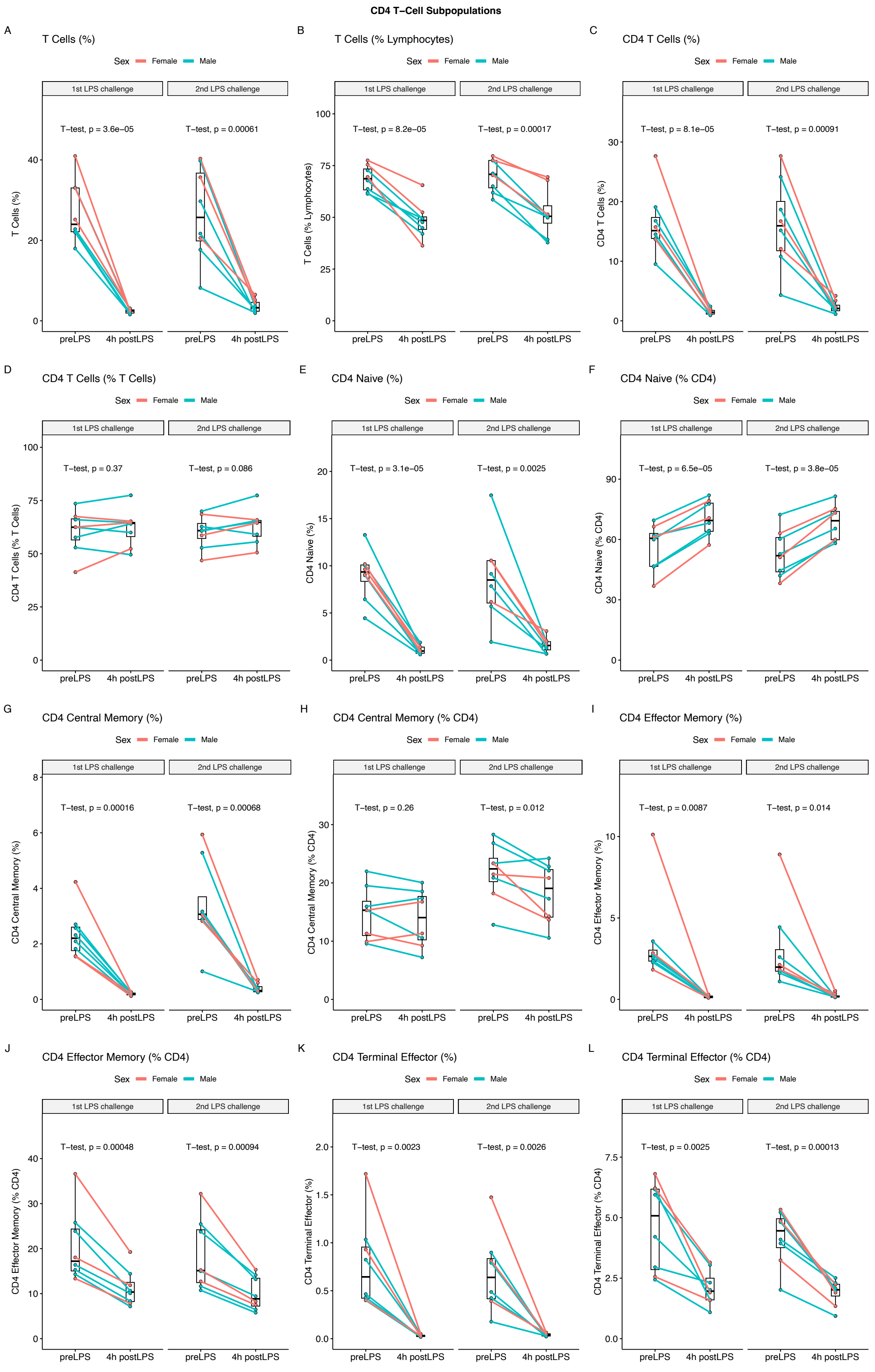

Supplementary Figure 9 CD4 T lymphocyte subpopulations before and 4h after LPS administration at the 1st and 2nd LPS challenge

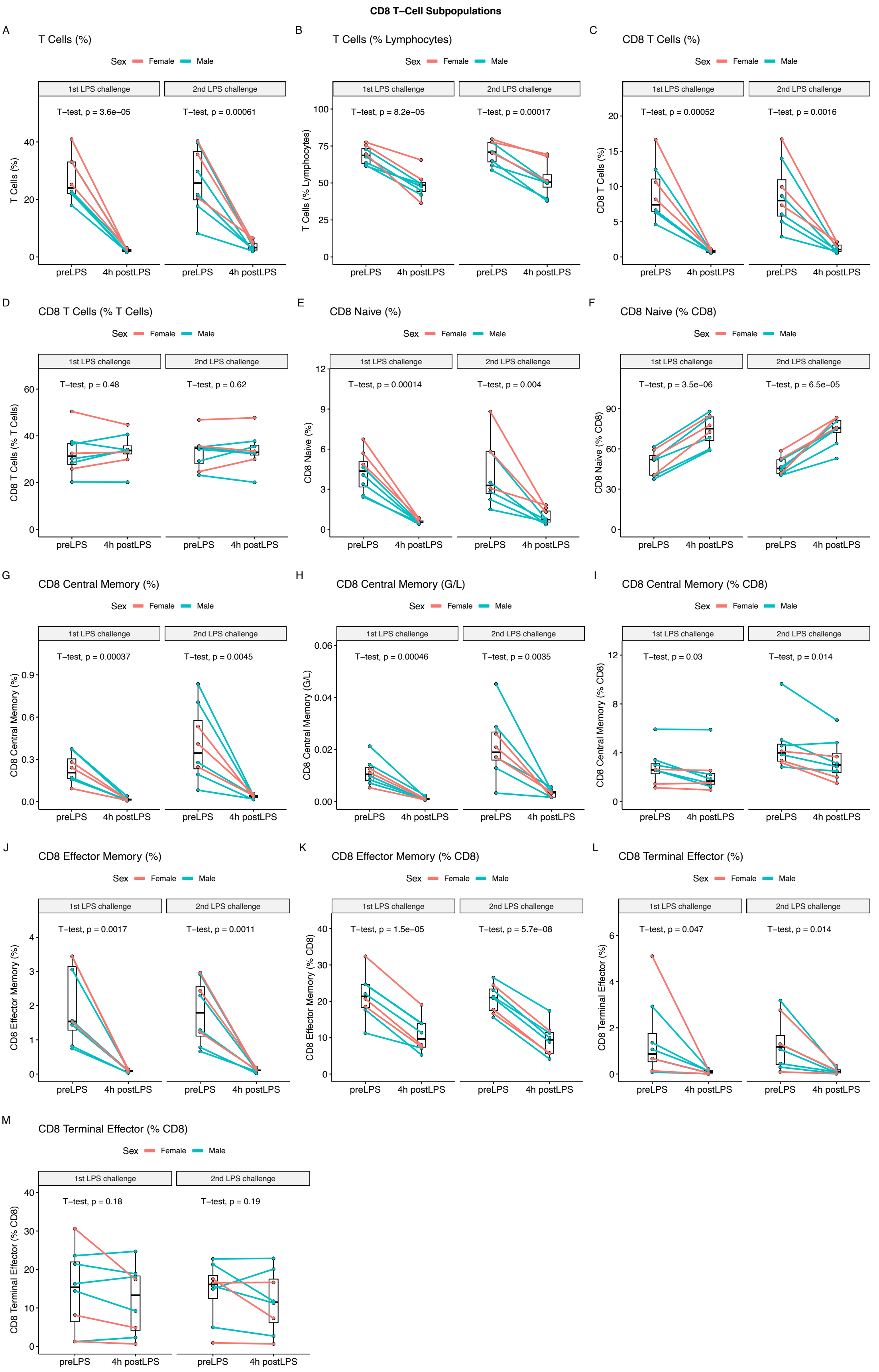

Supplementary Figure 10 CD8 T lymphocyte subpopulations before and 4h after LPS administration at the 1st and 2nd LPS challenge

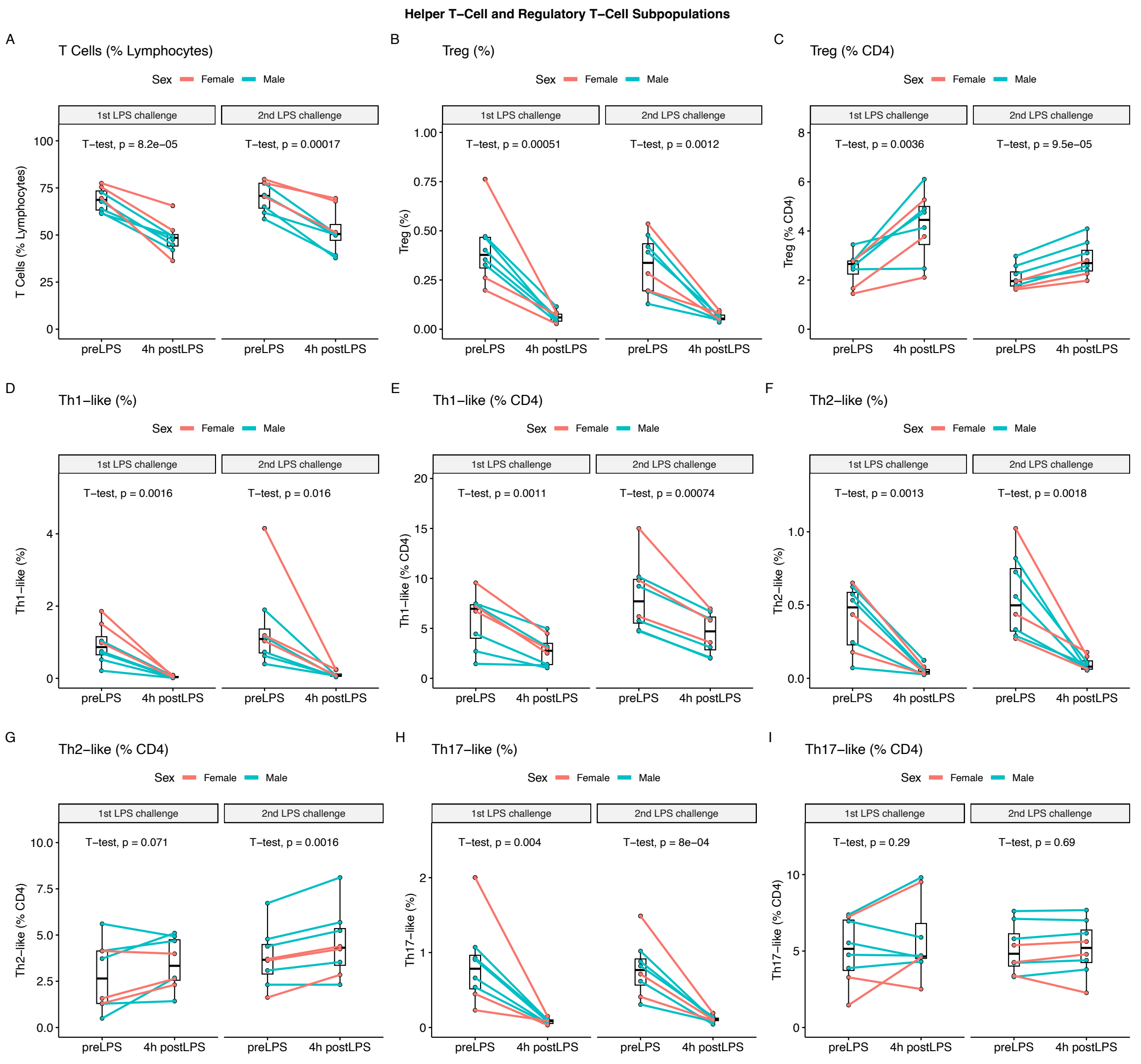

Supplementary Figure 11 Helper T lymphocyte and Regulatory T lymphocyte subpopulations before and 4h after LPS administration at the 1st and 2nd LPS challenge

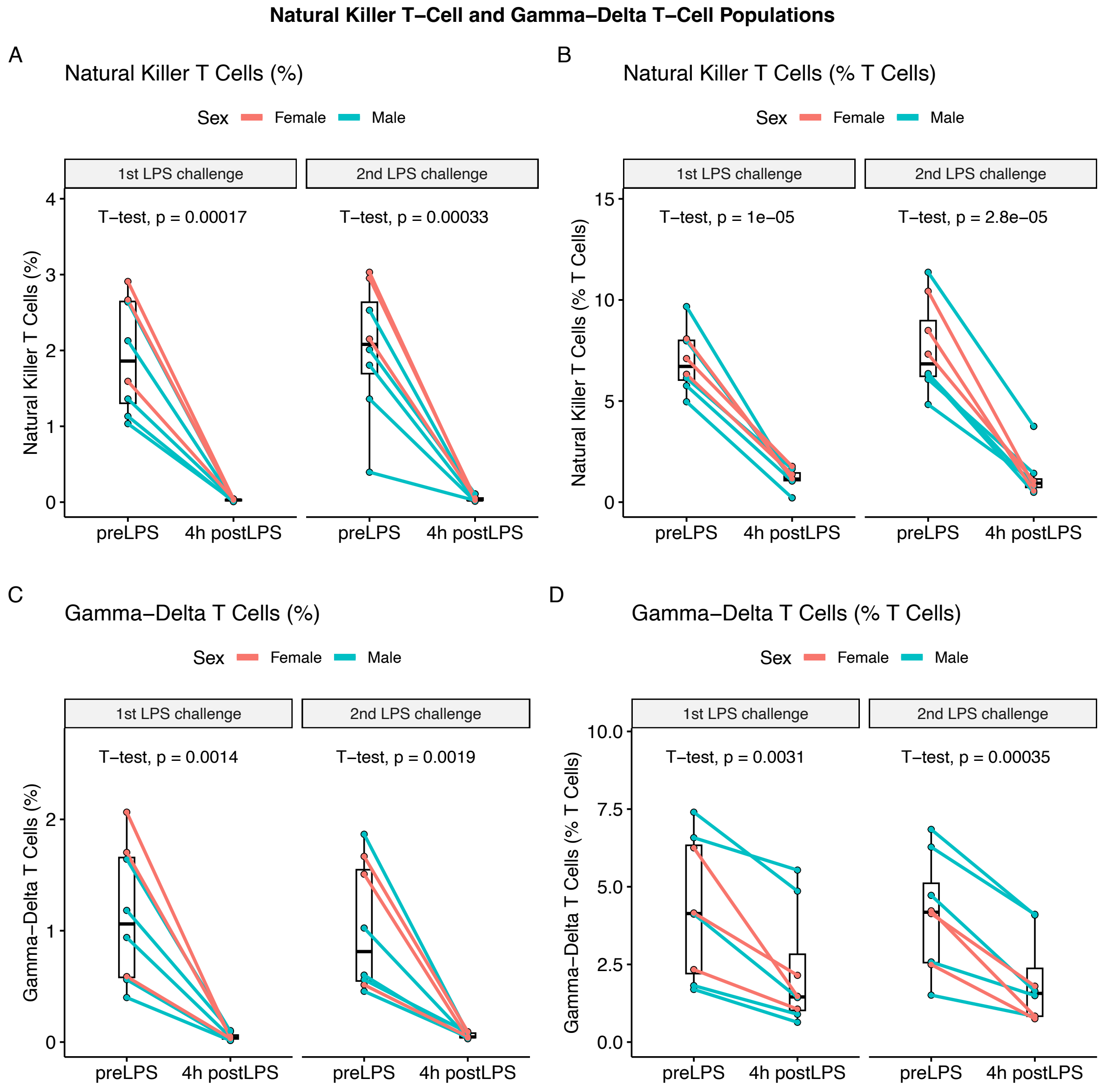

Supplementary Figure 12 Natural Killer T lymphocyte and Gamma-Delta T lymphocyte subpopulations before and 4h after LPS administration at the 1st and 2nd LPS challenge

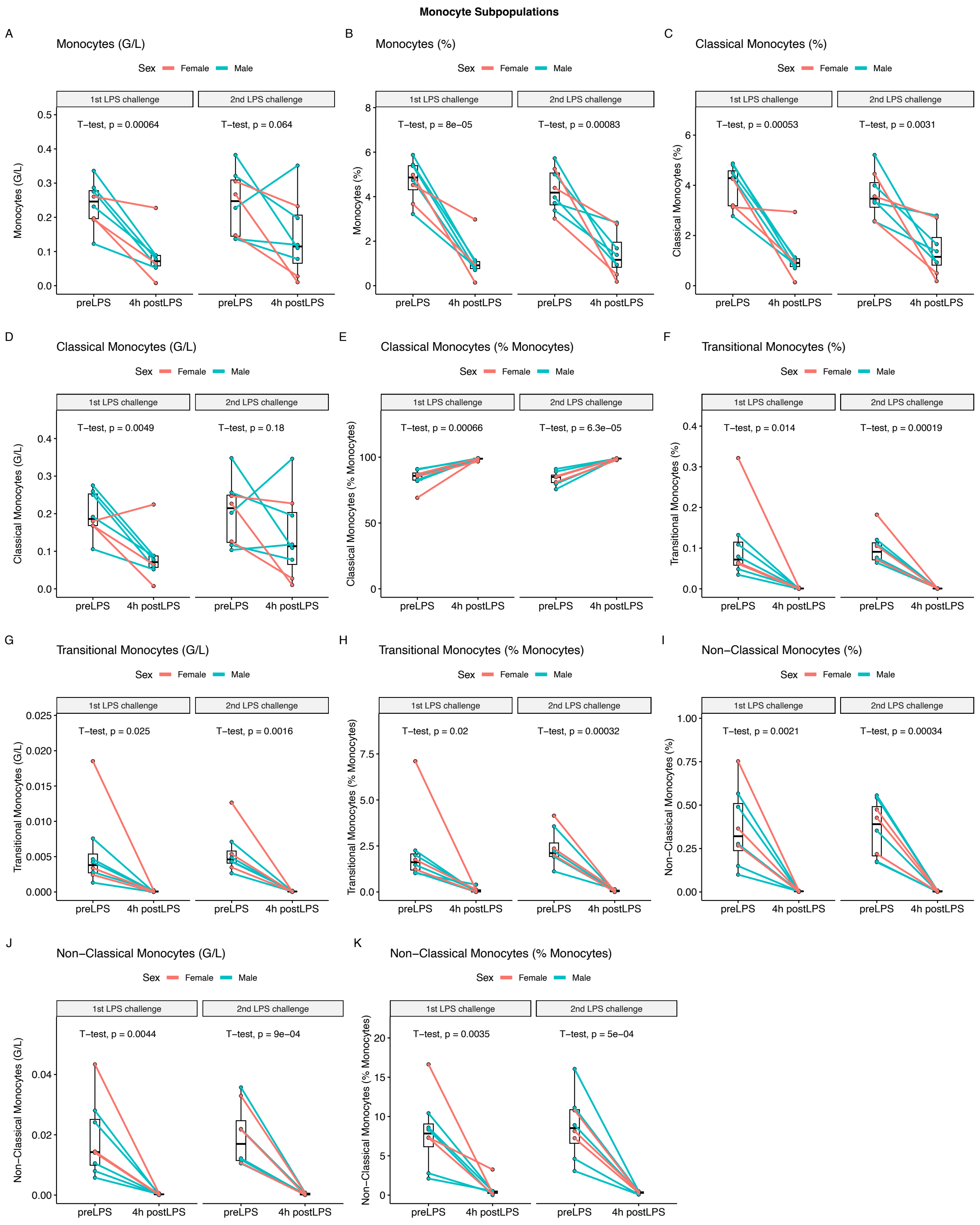

Supplementary Figure 13 Monocyte subpopulations before and 4h after LPS administration at the 1st and 2nd LPS challenge

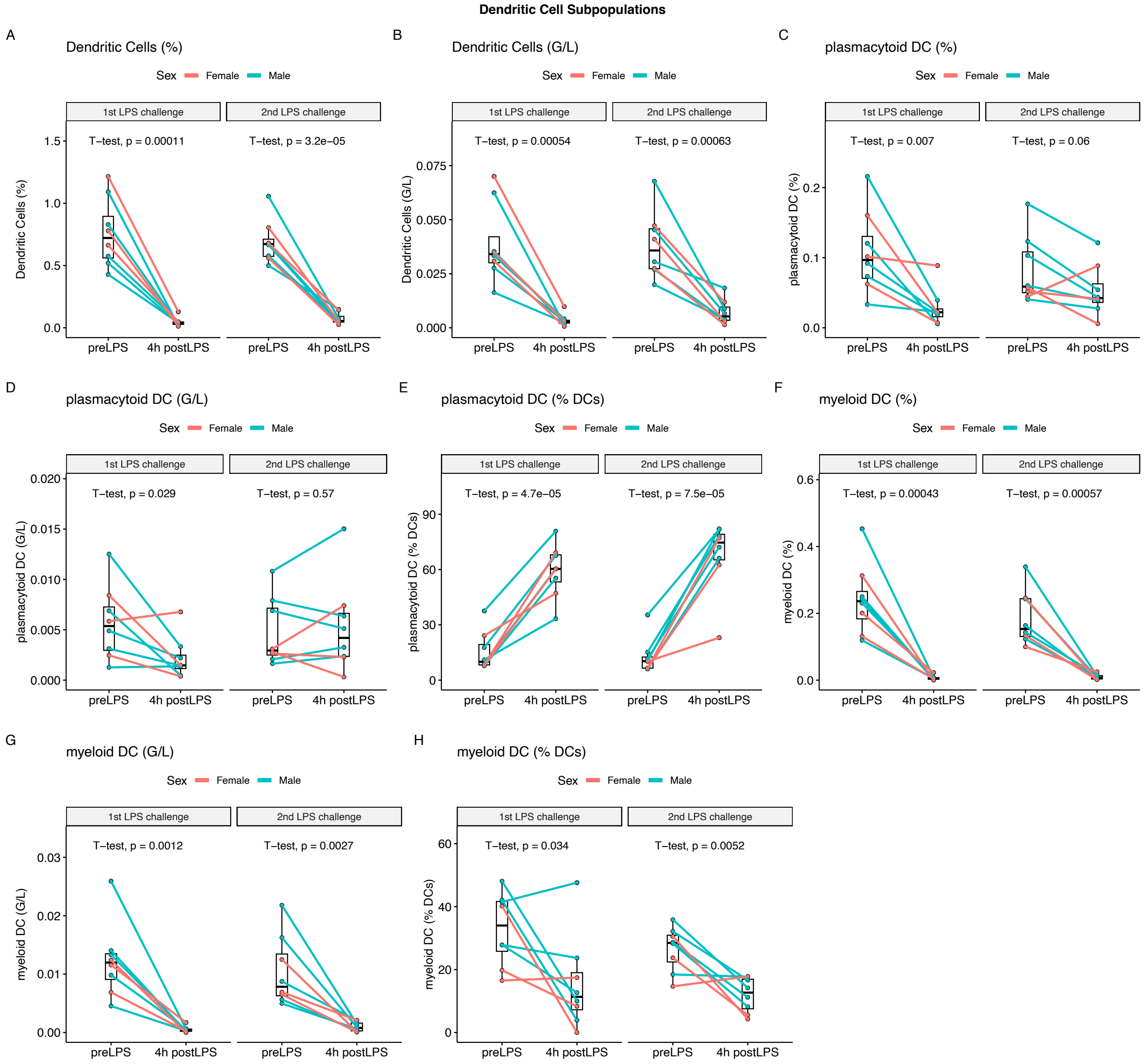

Supplementary Figure 14 Dendritic cell subpopulations before and 4h after LPS administration at the 1st and 2nd LPS challenge

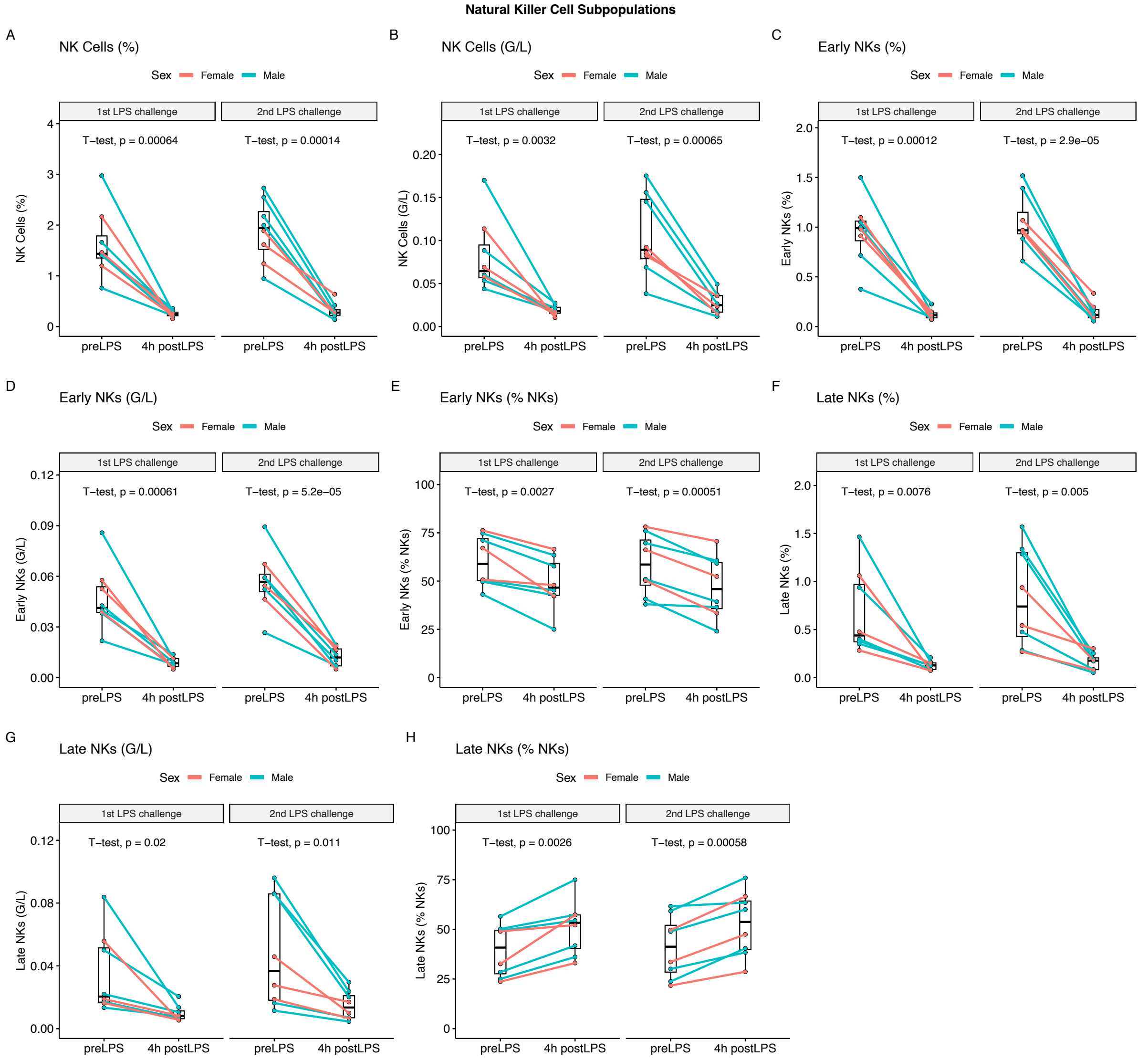

Supplementary Figure 15 Natural Killer cell subpopulations before and 4h after LPS administration at the 1st and 2nd LPS challenge

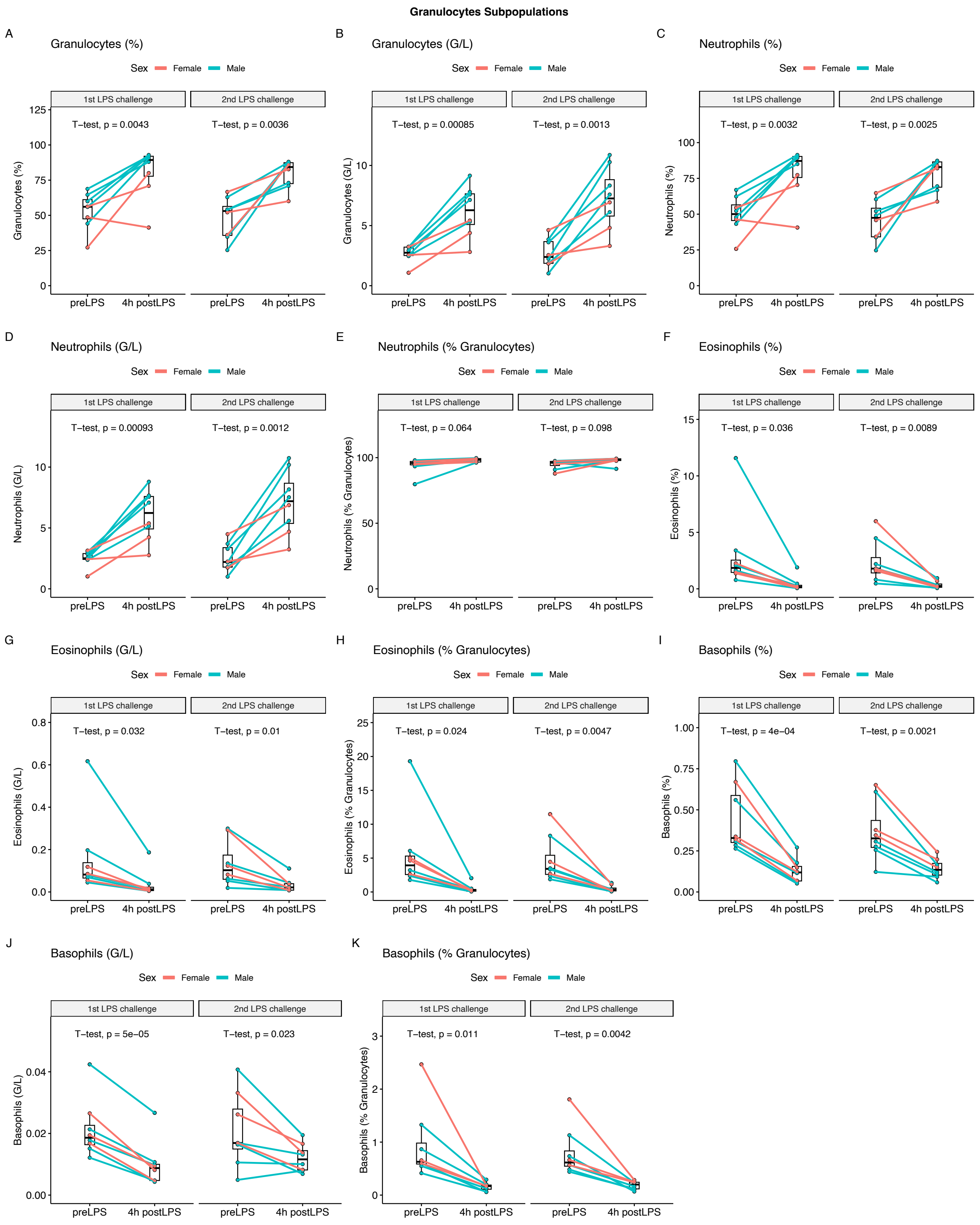

Supplementary Figure 16 Granulocyte subpopulations before and 4h after LPS administration at the 1st and 2nd LPS challenge

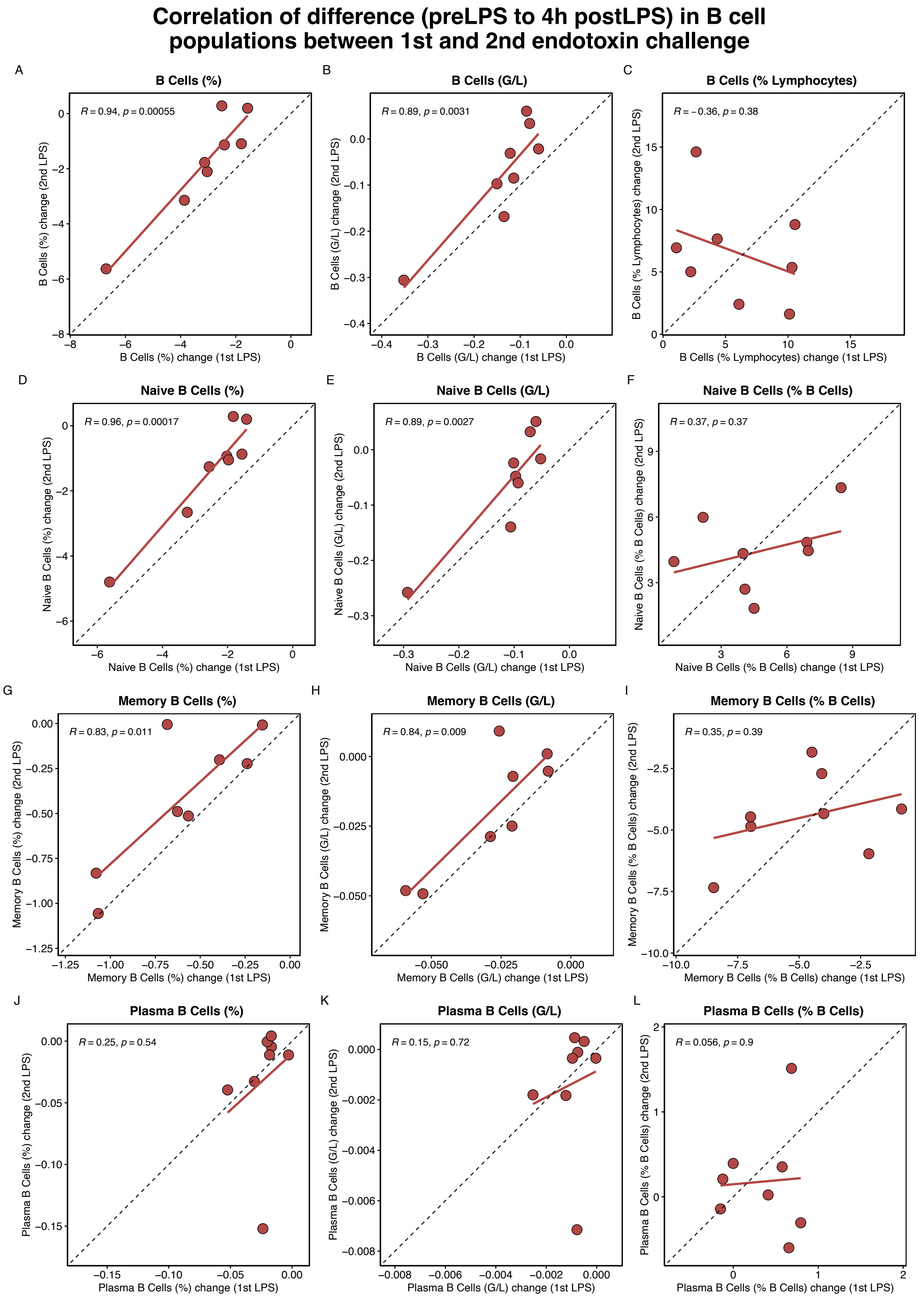

Supplementary Figure 17 Correlation of difference (Δ baseline to 4h) in B lymphocyte subpopulations between 1st and 2nd endotoxin challenge

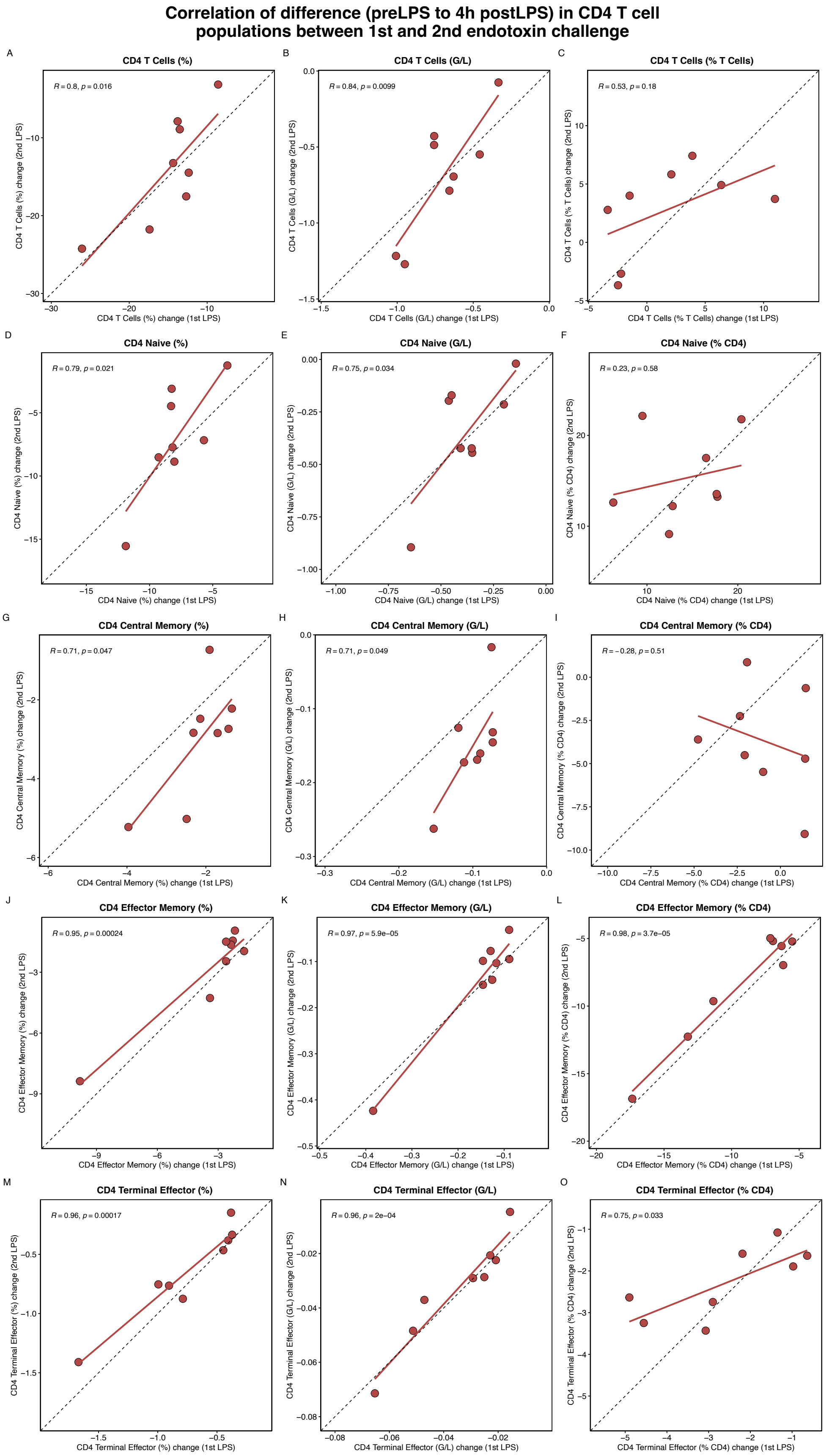

Supplementary Figure 18 Correlation of difference (Δ baseline to 4h) in CD4 T lymphocyte subpopulations between 1st and 2nd endotoxin challenge

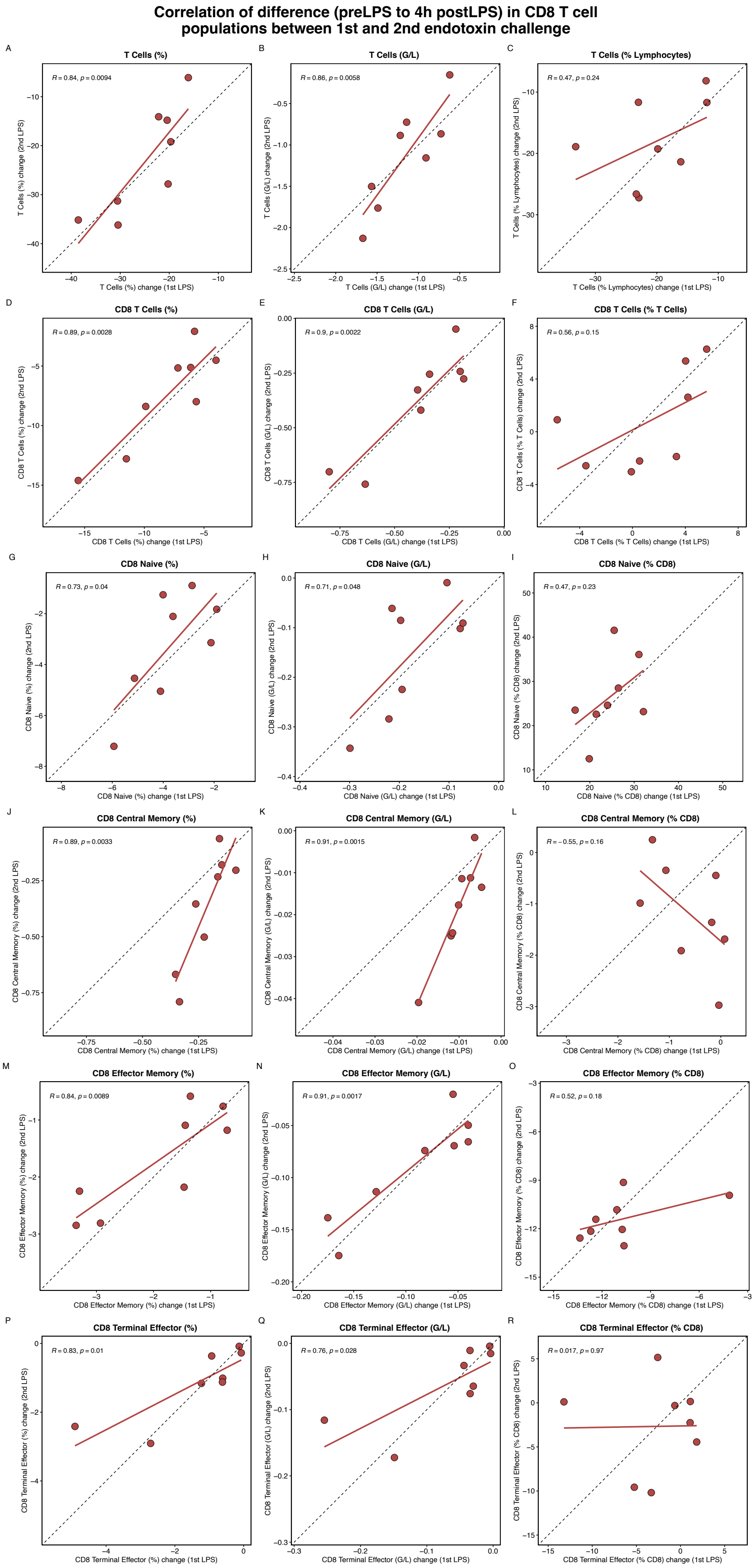

Supplementary Figure 19 Correlation of difference (Δ baseline to 4h) in CD8 T lymphocyte subpopulations between 1st and 2nd endotoxin challenge

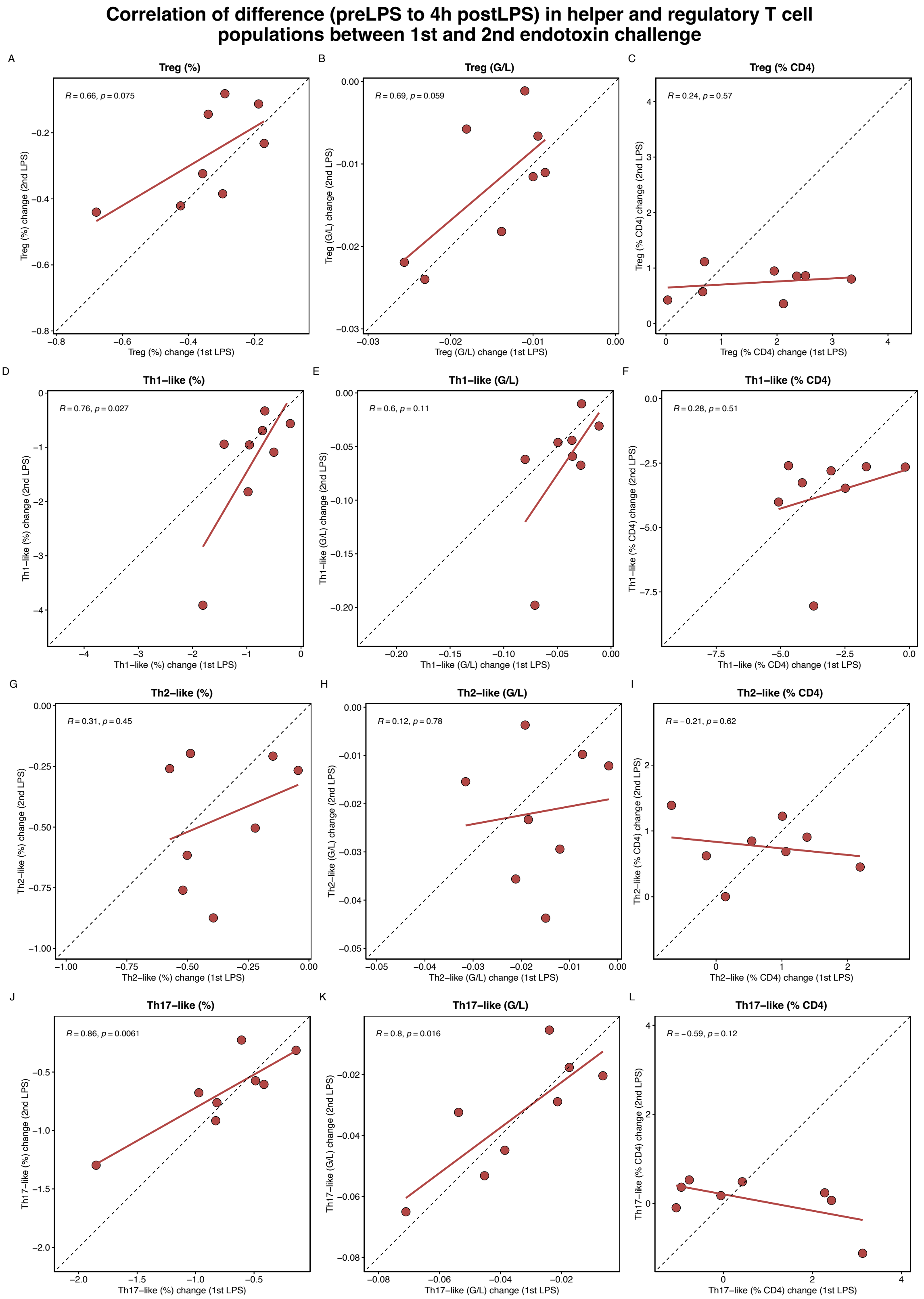

Supplementary Figure 20 Correlation of difference (Δ baseline to 4h) in helper and regulatory T lymphocyte subpopulations between 1st and 2nd endotoxin challenge

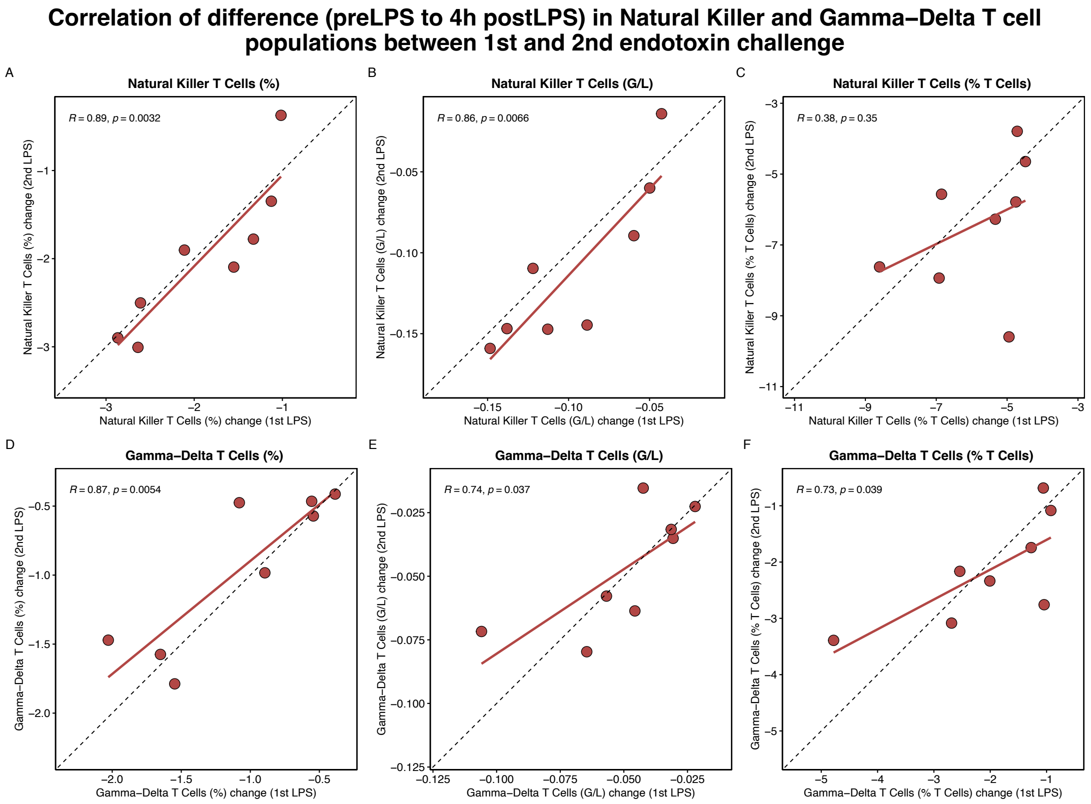

Supplementary Figure 21 Correlation of difference (Δ baseline to 4h) in Natural Killer T lymphocyte and Gamma-Delta T lymphocyte subpopulations between 1st and 2nd endotoxin challenge

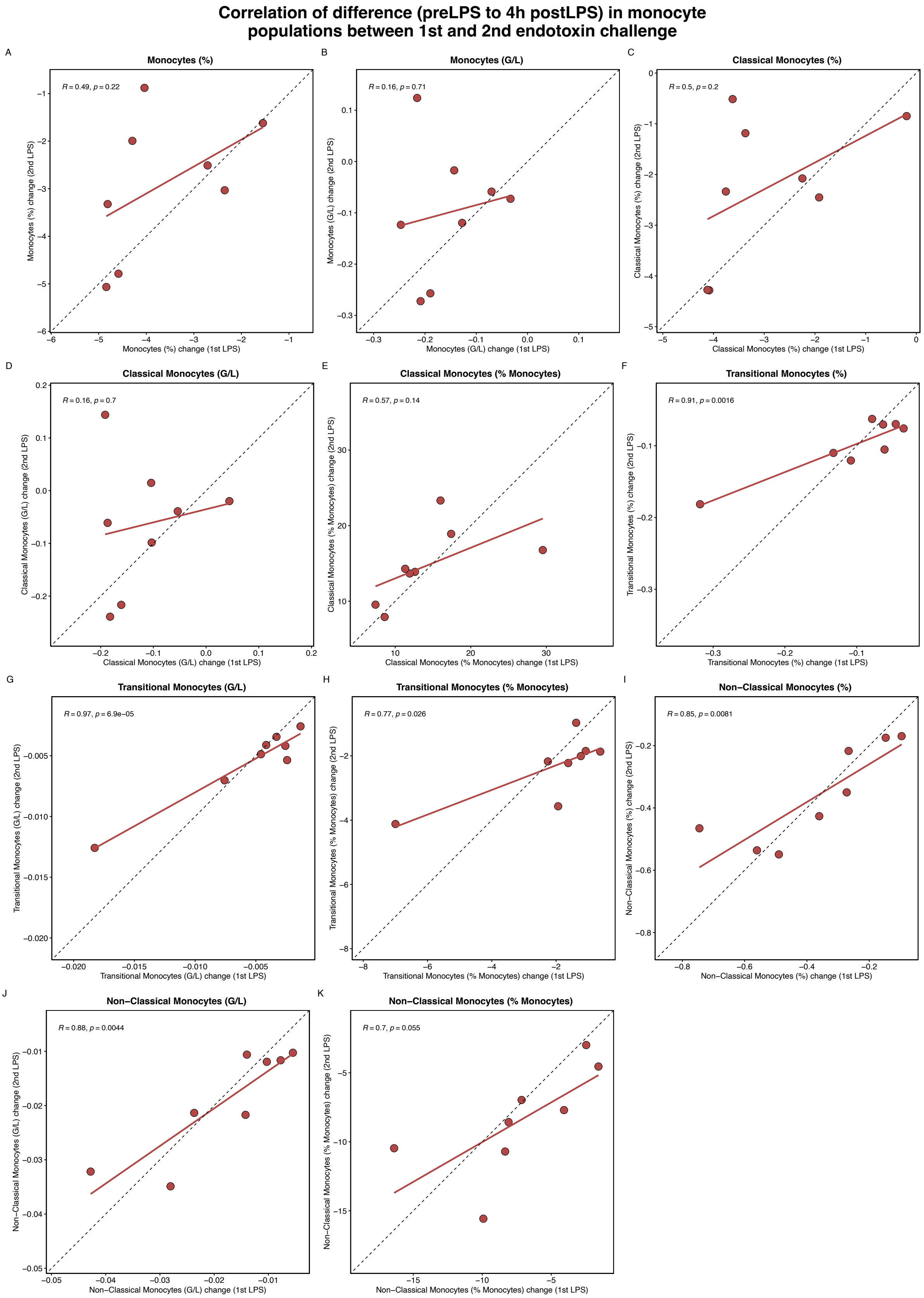

Supplementary Figure 22 Correlation of difference (Δ baseline to 4h) in monocyte subpopulations between 1st and 2nd endotoxin challenge

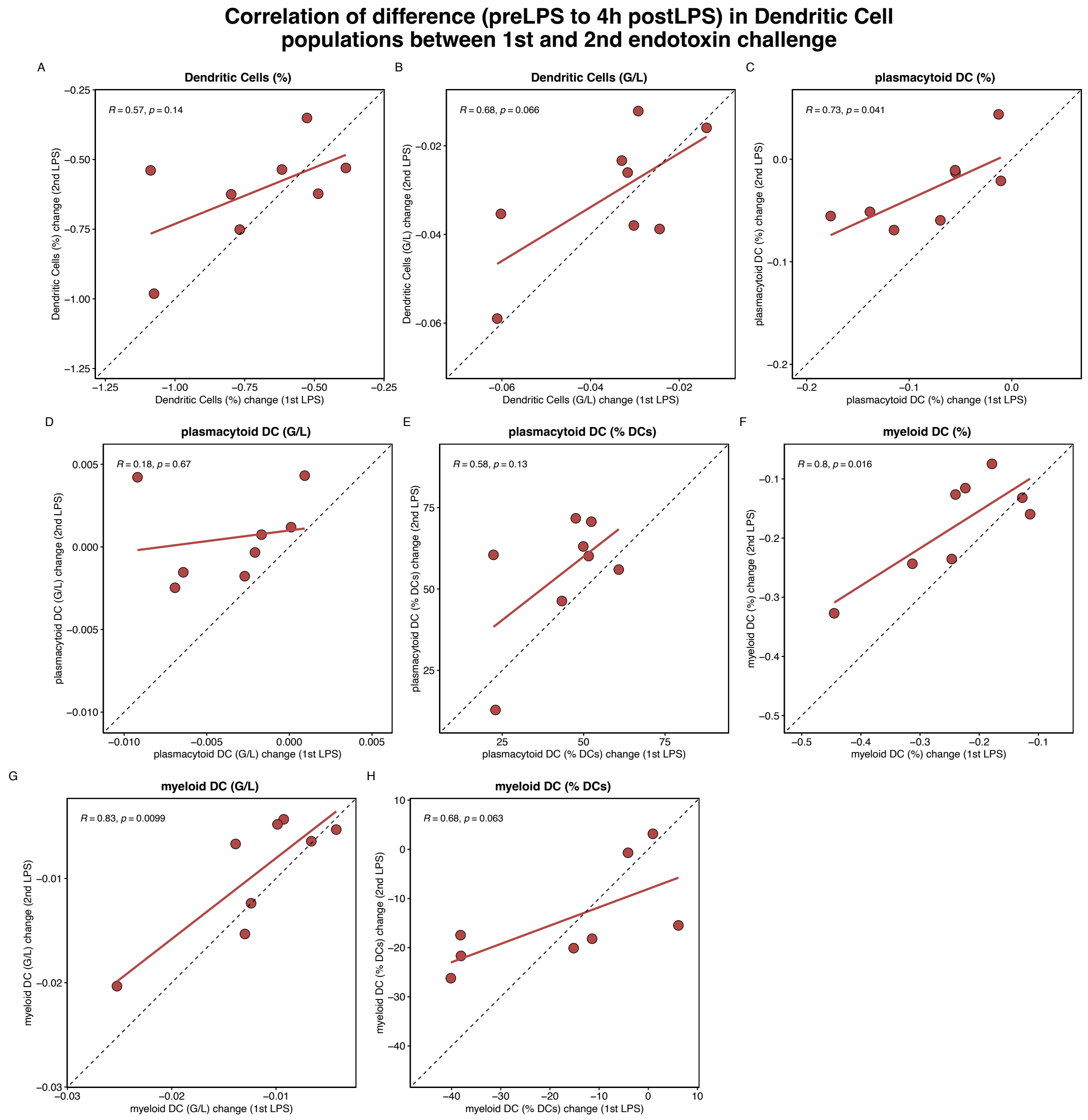

Supplementary Figure 23 Correlation of difference (Δ baseline to 4h) in dendritic cell subpopulations between 1st and 2nd endotoxin challenge

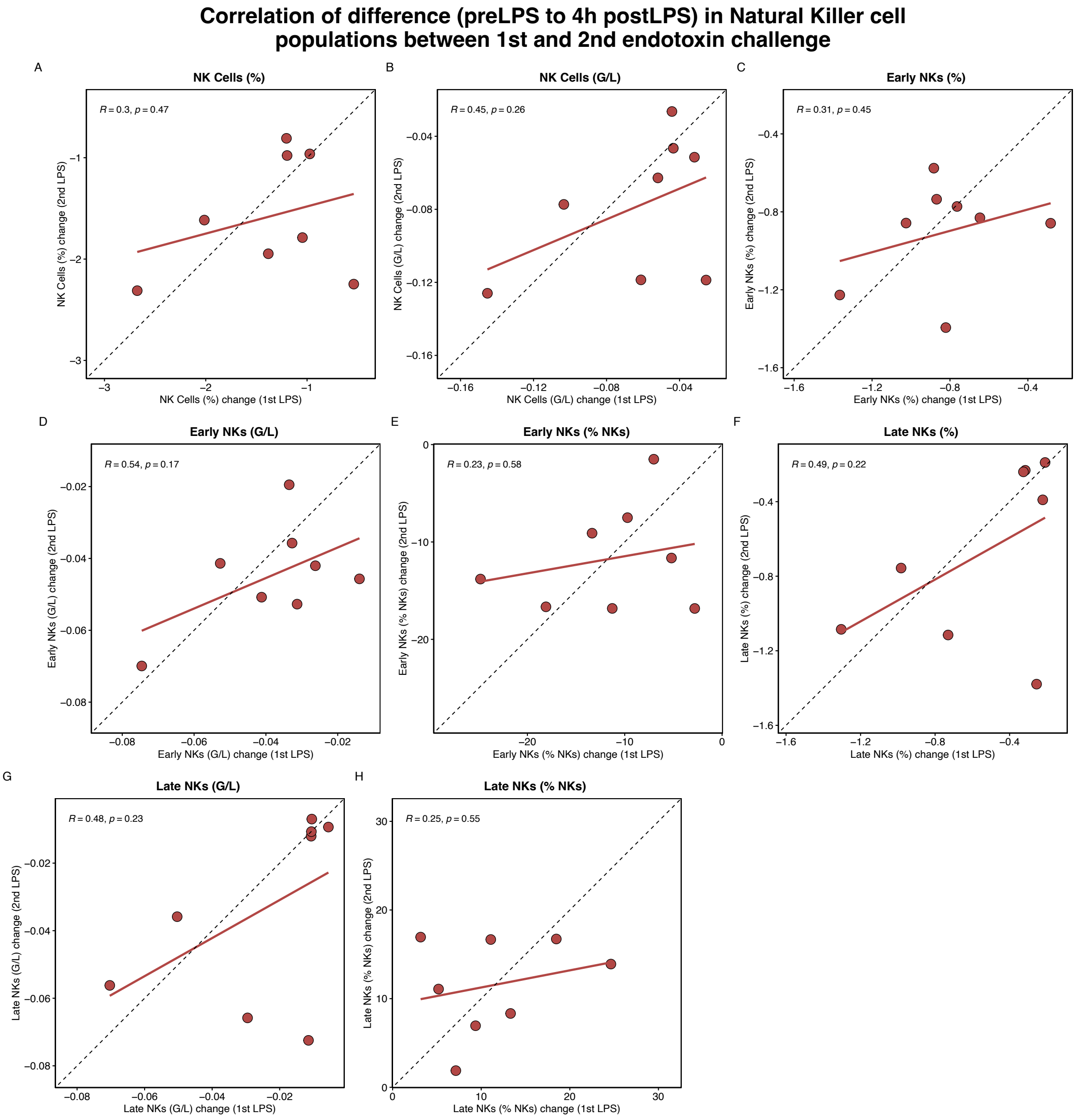

Supplementary Figure 24 Correlation of difference (Δ baseline to 4h) in natural killer cell subpopulations between 1st and 2nd endotoxin challenge

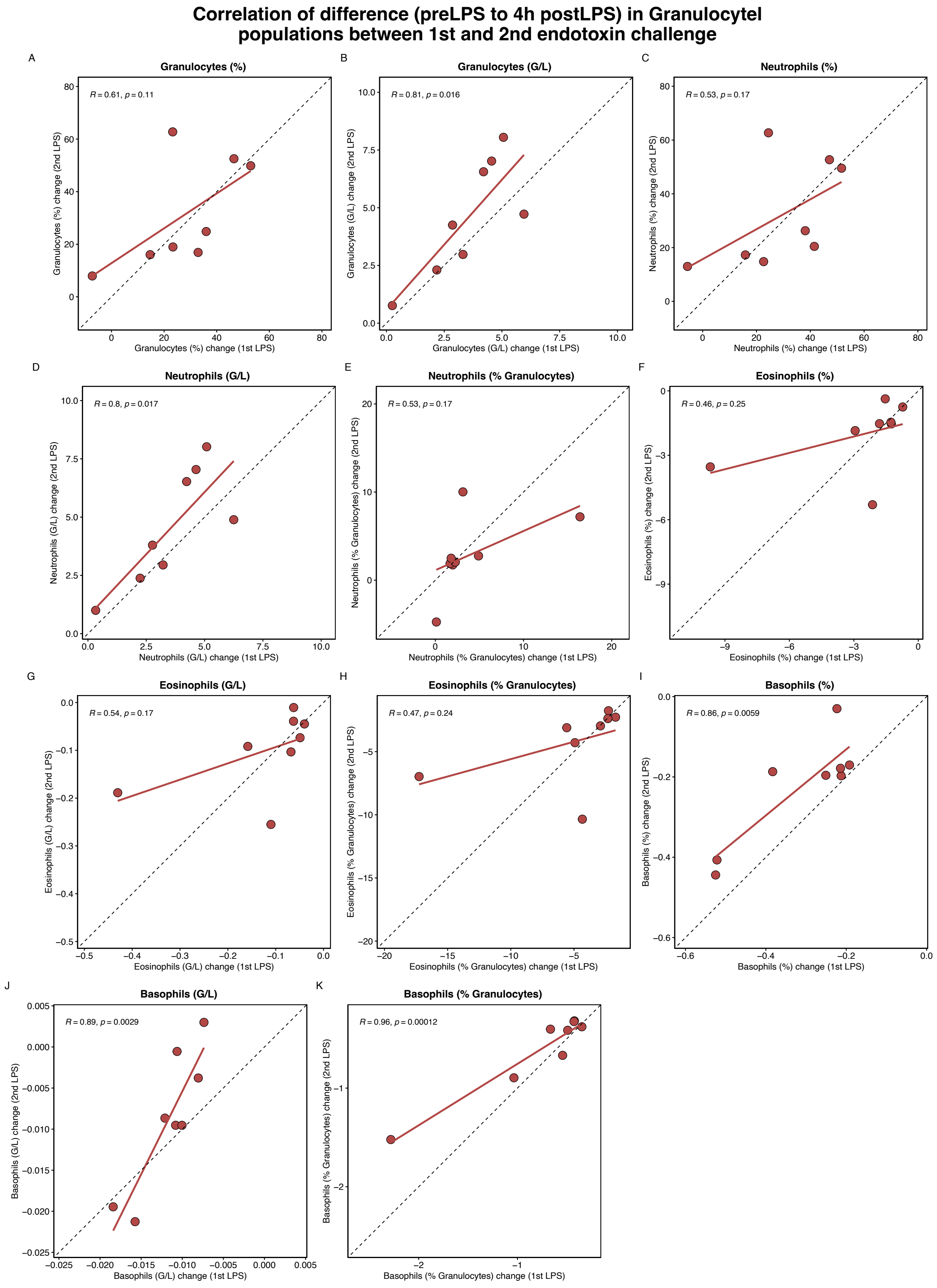

Supplementary Figure 25 Correlation of difference (Δ baseline to 4h) in granulocyte subpopulations between 1st and 2nd endotoxin challenge
